# Neuroendocrine transdifferentiation in human cancer: molecular mechanisms and therapeutic targets

**DOI:** 10.1002/mco2.761

**Published:** 2024-10-04

**Authors:** Jun Jiang, Donghui Han, Jiawei Wang, Weihong Wen, Rui Zhang, Weijun Qin

**Affiliations:** ^1^ Department of Urology Xijing Hospital Air Force Medical University Xi'an China; ^2^ Department of Health Service, Base of Health Service Air Force Medical University Xi'an China; ^3^ Department of Clinical Immunology, PLA Specialized Research Institute of Rheumatology & Immunology, Xijing Hospital, and National Translational Science Center for Molecular Medicine Air Force Medical University Xi'an China; ^4^ Xi'an Key Laboratory of Stem Cell and Regenerative Medicine, Institute of Medical Research Northwestern Polytechnical University Xi'an China; ^5^ State Key Laboratory of Cancer Biology Department of Immunology Air Force Medical University Xi'an China

**Keywords:** acquired resistance, lineage plasticity, neuroendocrine prostate cancer, neuroendocrine transdifferentiation, small cell lung cancer

## Abstract

Neuroendocrine transdifferentiation (NEtD), also commonly referred to as lineage plasticity, emerges as an acquired resistance mechanism to molecular targeted therapies in multiple cancer types, predominately occurs in metastatic epidermal growth factor receptor (EGFR)‐mutant non‐small cell lung cancer treated with EGFR tyrosine kinase inhibitors and metastatic castration‐resistant prostate cancer treated with androgen receptor targeting therapies. NEtD tumors are the lethal cancer histologic subtype with unfavorable prognosis and limited treatment. A comprehensive understanding of molecular mechanism underlying targeted‐induced plasticity could greatly facilitate the development of novel therapies. In the past few years, increasingly elegant studies indicated that NEtD tumors share key the convergent genomic and phenotypic characteristics irrespective of their site of origin, but also embrace distinct change and function of molecular mechanisms. In this review, we provide a comprehensive overview of the current understanding of molecular mechanism in regulating the NEtD, including genetic alterations, DNA methylation, histone modifications, dysregulated noncoding RNA, lineage‐specific transcription factors regulation, and other proteomic alterations. We also provide the current management of targeted therapies in clinical and preclinical practice.

## Graphical abstract

A schematic of the cell origin of SCLC and NEPC, including de novo and NEtD.

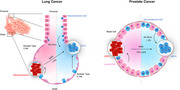

## 1 INTRODUCTION

Neuroendocrine transdifferentiation (NEtD) in human cancer represents a highly complex biological process, characterized by the lineage plasticity of epithelial cells. This process involves the transdifferentiation from an adeno‐lineage to a multilineage or stem‐like state, followed by a neuroendocrine (NE)‐lineage transition. NEtD is a mechanism of acquired resistance to targeted therapy, which predominately occurs in small cell lung cancer (SCLC) and NE prostate cancer (NEPC),^1^, ^2^ which transformed from metastatic epidermal growth factor receptor (EGFR)‐mutant non‐small cell lung cancer (NSCLC) treated with EGFR tyrosine kinase inhibitors (EGFR‐TKIs) and metastatic castration‐resistant prostate cancer (mCRPC) treated with secondary androgen deprivation therapies (ADTs) (e.g., enzalutamide), respectively.^3^ This process can be observed during histological analysis through typical morphological features such as high cell density, dense nuclei (high nuclear to cytoplasm ratios), frequent mitotic features (indicative of poor differentiation), granular chromatin, and the expression of typical NE markers, including synaptophysin (SYP), chromogranin A (CHGA), enolase 2 (ENO2, NSE), neural cell adhesion molecule 1 (NCAM1, CD56), insulinoma‐associated protein 1 (INSM1), and so on.^4^ In vitro, NE cancer cells typically exhibit a neuronal morphology with rounded cell bodies and finely bifurcated extensions.

**FIGURE 1:**
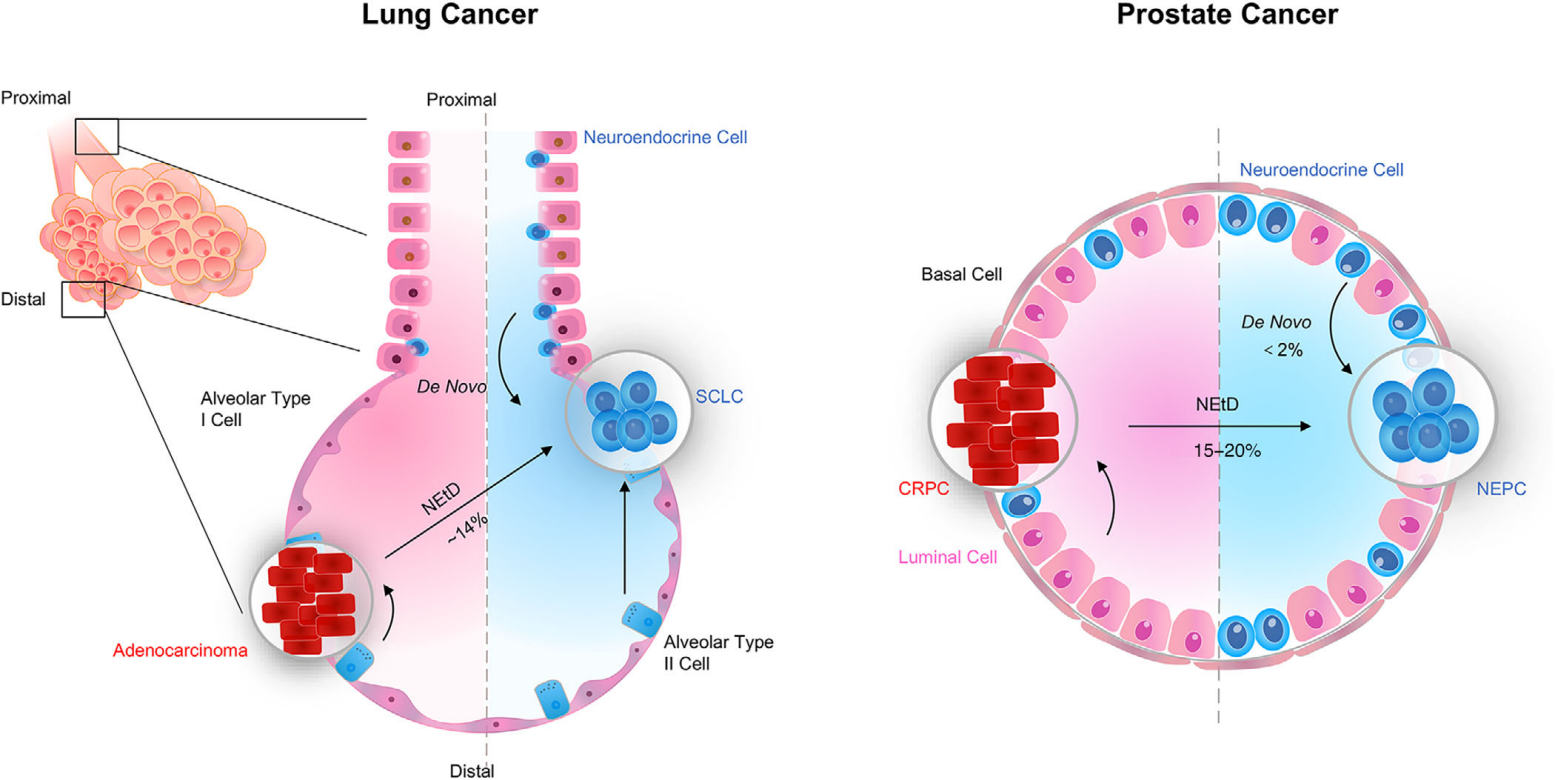
A schematic representation of the cell origin of SCLC and NEPC, including resident neuroendocrine cell (de novo) and lineage plasticity (NEtD). Lung adenocarcinoma mainly arise from pulmonary alveolar type II cell in alveolus (distal airways), whereas SCLC arise from proximal and resident neuroendocrine cell.^202^ These anatomical features account for that adenocarcinomas are mainly peripheral lung cancer whereas SCLCs are central lung cancer. Additionally, SCLC also can origin from alveolar type II cell or adenocarcinoma NEtD (∼14%) driving by molecular events (loss of TP53, RB1, and other genetic, epigenetic alterations). Similarly, de novo NEPC is rare (<2%), NEPC are mainly derived from the NEtD of luminal epithelial cells (15–20%), rarely origin from resident and interspersed neuroendocrine cells.

NEPC, a highly aggressive CRPC variant independent of androgen receptor (AR) signaling, has been documented in 15−20% of CRPC cases following AR inhibitor treatment^5^ (Figure 1). Similarly, approximately 14% of lung adenocarcinoma (LUAD) patients undergo SCLC transformation^6^ (Figure 1). Nevertheless, de novo NEPC is rare, occurring in less than 2% of cases.^7^ NEPC is characterized by a decrease in luminal epithelial markers (cytokeratins [CK] 8 and 18) and an increase in basal epithelial markers (CK 5 and 14), along with the expression of typical NE markers.^8^ NEtD tumors are typically treatment‐refractory and rapidly progressive, associated with a poor prognosis similar with or worse than that of de novo NE carcinoma.^5^, ^9^

**FIGURE 2:**
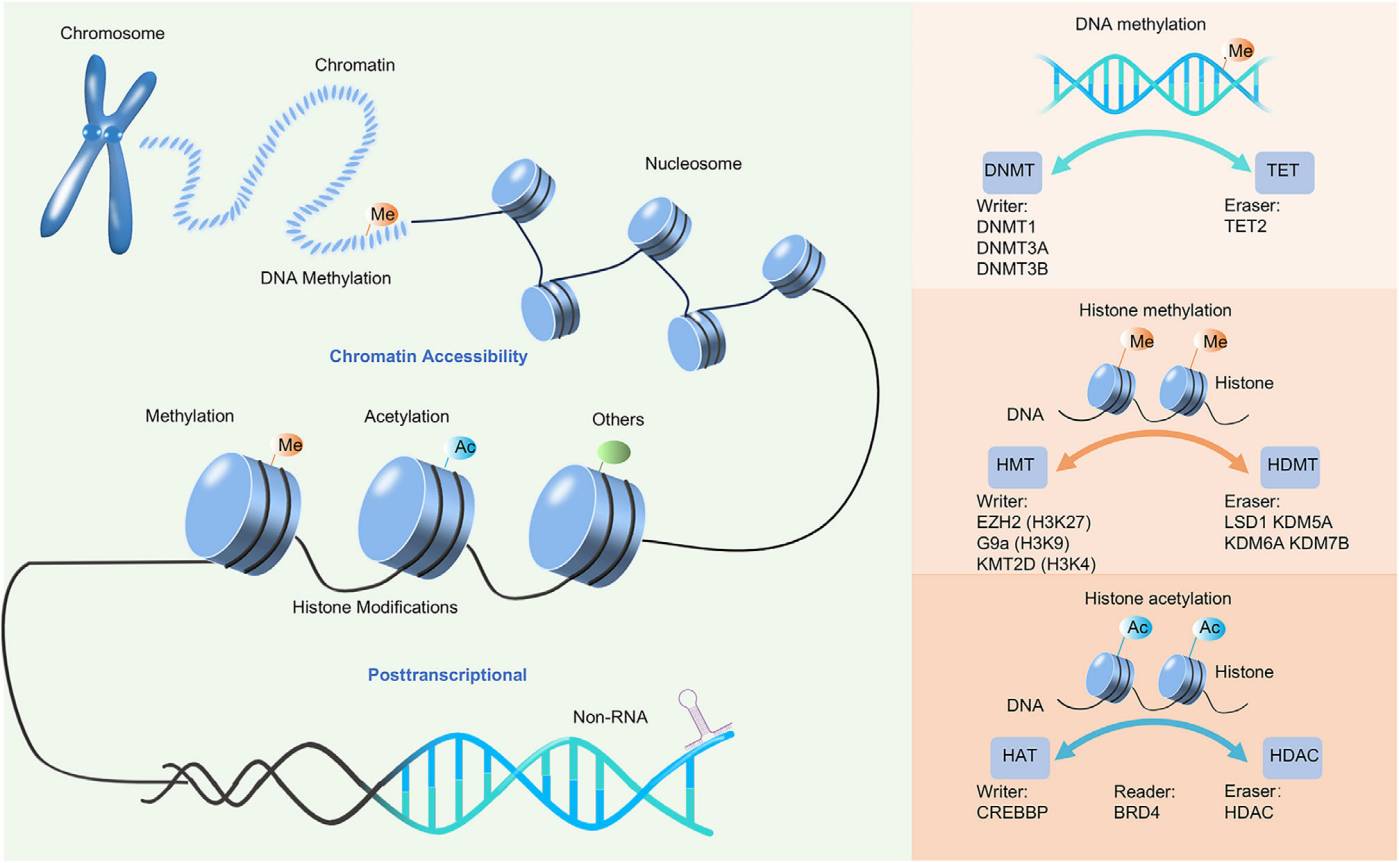
The overview of epigenetic regulation of NEtD. Epigenetic regulation mainly includes DNA methylation, histone modifications (methylation, acetylation, and others) and noncoding RNAs. Hypermethylation of CpG islands in promoter region of tumor suppressors genes is catalyzed by DNA methyltransferases (DNMTs) and removed by ten eleven translocations (TETs). Methyl‐groups are catalyzed by histone methyltransferases (HMTs) and removed by histone methyltransferases (HDMTs). Acetyl‐groups are catalyzed by histone acetyltransferases (HATs), removed by histone deacetylases (HDACs), recognized by BETs family.

**FIGURE 3:**
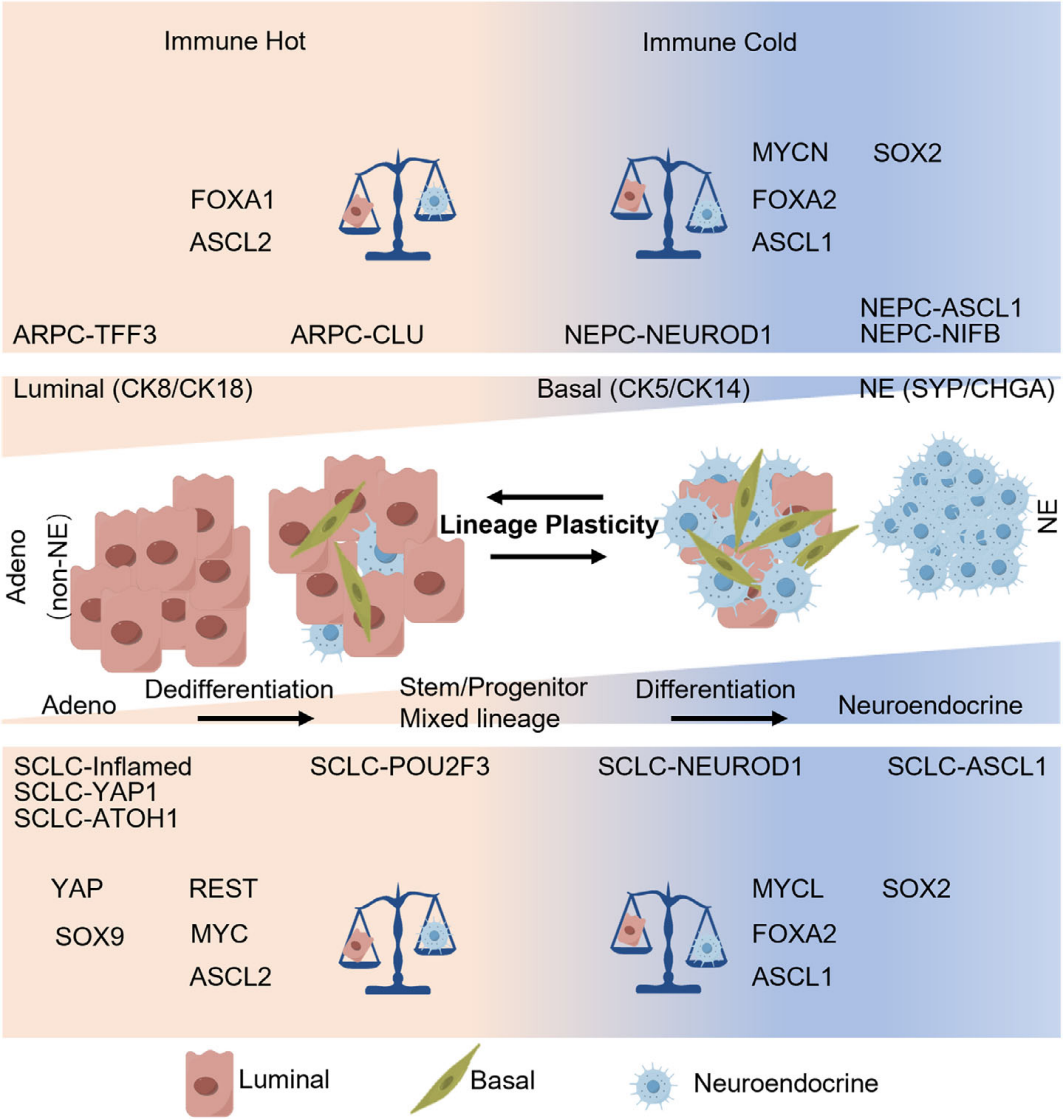
The lineage plasticity and molecular subtypes of SCLC and NEPC based on lineage pioneering transcription factors (TFs). Epithelial cell fate is determined by pioneering TFs. FOXA2, ASCL1, SOX2 drives NEtD with neuroendocrine markers upregulation, while FOXA1, ASCL2, SOX9 drives non‐neuroendocrine lineage development. NOTCH/REST and YAP signaling are enriched in non‐NE–SCLC and suppress the process of NEtD. Based on molecular heterogeneity, SCLC can be divided into ASCL1, NEUROD1, POU2F3, and the fourth controversial subtype (YAP, ATOH1, or inflamed). Similarly, mCRPC also can be divided into NE–CRPC (NEPC) and non‐NE CRPC. During the process of lineage plasticity with targeted therapies selective pressure, prostate adenocarcinoma cancer cells gradually dedifferentiate and lost luminal features (CK8, CK18), acquire basal cell (CK5, CK14), and stem cell characteristics (SOX2) with mixed lineage concurrence, then further differentiate into NE lineage and obtain NE markers (SYP, CHGA, ENO2/NSE, and NCAM1/CD56). The distinct molecular subtypes also confer NEtD tumors with differential response to immunotherapy. The non‐NE–SCLC subtypes enriched in inflamed genes like MCH‐I are relatively responsive to immune checkpoint inhibitors. NE, neuroendocrine; Adeno‐, adenocarcinoma.

The advent of technologies such as whole‐exome sequencing (WES), CRISPR systems (knock in or out), single‐cell multiomics, genetically engineered mouse models (GEMM), organoids culture, and PDX preclinical models has facilitated the systematic delineation of genomic, epigenetic, and transcriptomic atlases in the molecular mechanism of NEtD. Despite originating from different organs and distinct epithelial tissues, NEtD share typical genetic and epigenetic features to some extent.^10^ For example, both SCLC and NEPC exhibit concurrent genetic loss of tumor suppressors RB1 and TP53, as well as mutually exclusive expression of C‐MYC (MYC), N‐MYC (MYCN) or L‐MYC (MYCL).^11^ Specifically, MYCL has been associated with NEtD in SCLC, while MYC drives non‐NEtD in SCLC, and MYCN is essential for NEPC development. Epigenetic regulation (Figure 2), including DNA methylation (DNA methyltransferase (DNMT)) and histone modifications (such as enhancer of zeste homolog 2 [EZH2]), plays a significant role in NEtD. Moreover, lineage‐specific transcription factors (TFs) play a crucial role in determining lineage bifurcation (Figure 3), with SCLC divided into four molecular subtypes, including ASCL1, NEUROD1, TF POU class 2 homeobox 3 (POU2F3), and YAP1, or Inflamed subtype, similar with the mutually exclusive pattern of the MYC family.^12^, ^13^ CRPC can also be classified into ASCL1/NEUROD1 (NE) subtype and non‐ASCL1/NEUROD1 (non‐NE) subtype.^14^ Moreover, multiple temporal studies based on preclinical models have identified TFs such as ASCL1/2, FOXA1/2, and SOX2/9 TFs switch as key regulators in distinct transdifferentiation trajectories.^15^, ^16^ For instance, ASCL1 serves as a pioneering NE lineage‐specific TF in both NEPC and SCLC,^12^, ^15^, ^17^ while ASCL2 produces the non‐NE lineage in lung cancer^18^ and the luminal lineage (non‐NE) in prostate cancer.^15^

Understanding the distinct histological transitions, cellular heterogeneity and dynamic molecular events during lineage plasticity can aid in personalized treatment and the development of therapeutic targets to improve patient prognosis. This review aims to summarize and provide multiomics insights into the mechanisms of NE lineage plasticity development in the process of NEtD, particularly in SCLC and NEPC, encompassing genetic mutations, epigenetic variabilities, TFs, and proteomic alterations. We also highlight promising genetic and epigenetic‐targeted inhibitors alone or in combination strategies for refractory NEtD tumors.

## 2 GENOMIC ABERRATIONS

**FIGURE 4:**
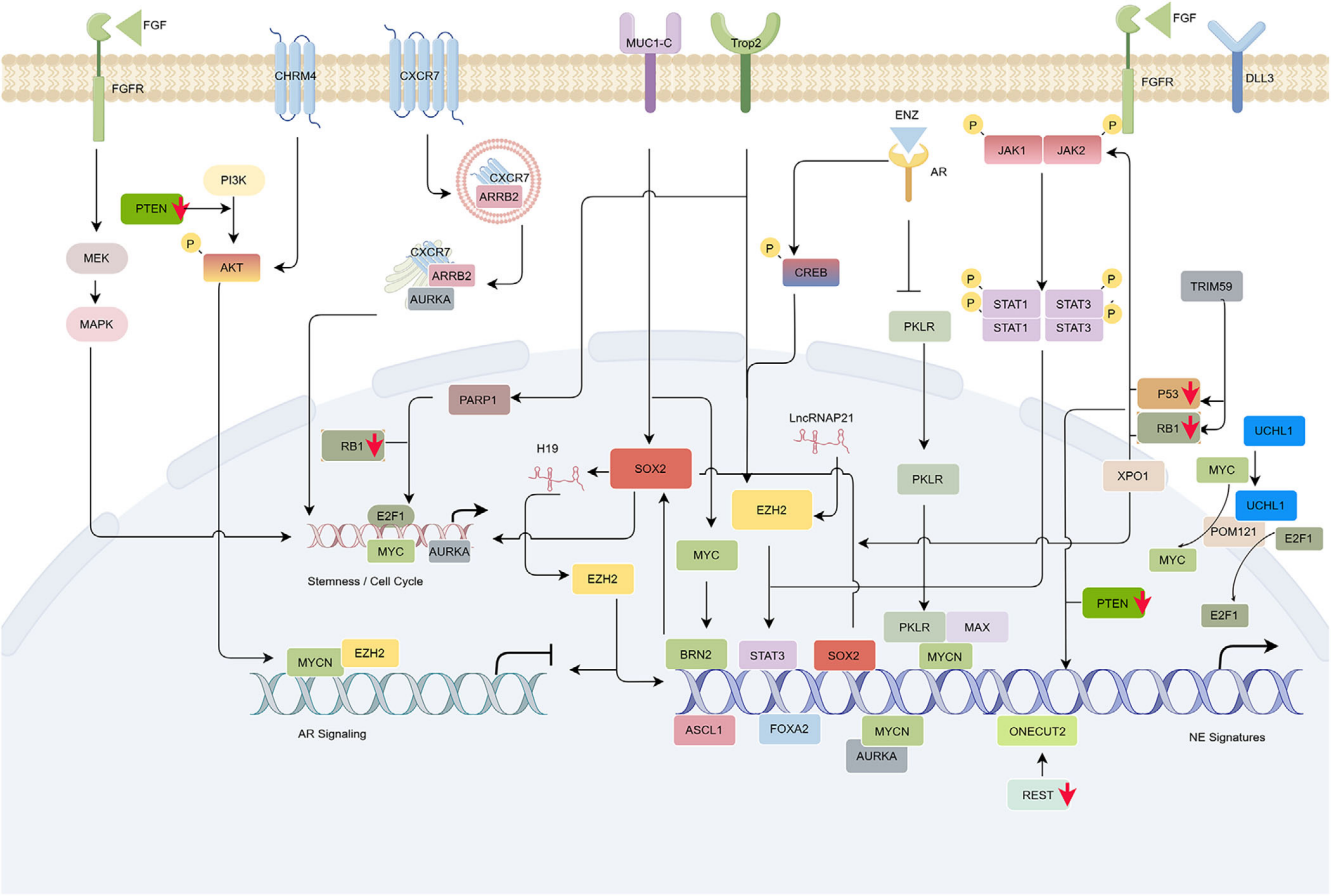
Schematic overview of pathways driving NEtD in NEPC. Molecular alterations regulate the lineage plasticity through agitating multiple pathways anticipate in including activations of PI3K/AKT, JAK/STAT and cell cycle (E2F1, SOX2, MYC, and AURKA) signaling, and AR signaling loss. RB1 loss, leading to E2F1 upregulation, drive NEtD initiation with TP53 mutation by activating SOX2, which can be enhanced by PTEN loss. PTEN loss also activated AKT signaling, driving downstream activation including mTOR, MYC, and AURKA which promote cell cycle signaling. Cell membrane proteins (MUC1‐C, TROP2, CHRM4, CXCR7, and FGFR) overexpression and activation transmit survival and NE differentiational signals via above pathways. Nuclear membrane proteins (POM121 and XPO1) facilitate the nuclear transportation of oncogenic proteins.

**FIGURE 5:**
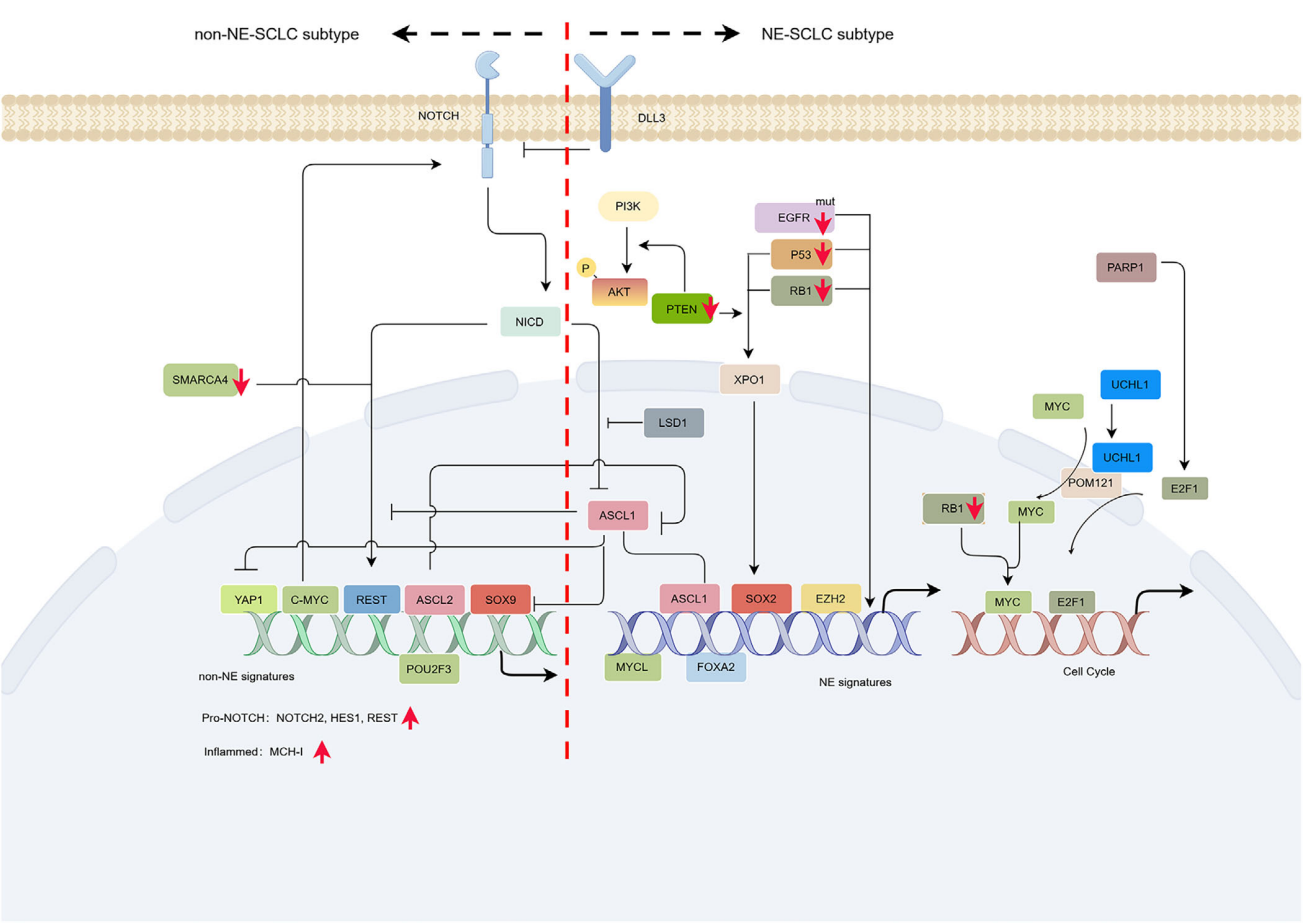
Schematic overview of pathways regulating lineage plasticity in distinct SCLC subtypes. NOTCH signaling activation and SMARCA4 loss promote non‐NE subtype development, whereas AKT signaling activation and Notch signaling loss (DLL3 upregulation) driving NEtD. PTEN loss facilitates the initiation of NE–SCLC driving by TP53 and RB1 alterations. Additionally, concurrent alterations of RB1 and TP53 promote EGFR‐mutant lung cancer transform to SCLC. Secreted protein UCHL1 maintains NEtD by regulating nucleoporin POM121, which mediates nuclear transport of E2F1 and MYC.

Given the emergence of WES, high mutational burden and molecular alterations in NEtD tumors were gradually characterized. Accumulating evidence displaying driver alterations including somatic mutations and change in copy numbers is required in driving lineage plasticity and tumor progression in NEPC (Figure 4) and SCLC (Figure 5).

### 2.1 RB1/TP53 loss

The loss of RB1/TP53 is ubiquitous in NEPC^19^, ^20^ and SCLC.^9^, ^21^ In 2017, two studies published in “Science” revealed that the loss of TP53/RB1 promotes the transition to NE‐lineage by increasing the expression of SOX2 and EZH2. It was further demonstrated that restoring TP53/RB1 and inhibiting SOX2 could reverse the NEtD process in prostate cancer.^19^, ^20^ WES data from surgical resection and biopsy specimens of metastatic prostate cancer indicate a significantly elevated probability of simultaneous RB1 deficiency and TP53 alterations in NEPC compared with adenocarcinoma. RB1 loss has been documented in over 70% of NEPC patients, with 66.7% having TP53 mutations or deletions. Furthermore, more than 50% of patients exhibit both mutations concurrently, contrasting with the 14% observed in CRPC patients.^22^

Similarly, a real‐world cohort study comprising 3600 SCLC patients revealed that 91.6% exhibited TP53 deletion or mutation, and 73.5% displayed RB1 deletion.^23^ The significance of TP53/RB1 inactivation in triggering both SCLC and NEPC has been substantiated and extensively utilized in genetically engineered mice models for temporal evolution research.^10^, ^15^, ^24^

In addition, inactivation of TP53/RB1 is also the most frequently in small cell bladder cancer, each arising in 90% of patients.^25^ Taken together, the loss of TP53/RB1 represents the most prevalent type of mutation in NE phenotype irrespective of their site of origin, already present at the inception of the primary tumor, serving as a crucial step in initiating NEtD.

### 2.2 Phosphatase and tensin homolog deleted on chromosome 10 loss

Phosphatase and tensin homolog deleted on chromosome 10 (PTEN), a potent tumor suppressor, represents a common and clinically relevant genetic mutation observed across various malignancies. In addition to TP53/RB1 mutations driving NEtD, the loss of PTEN also plays a crucial role in shaping the evolution of lethal NE tumor types.^26^ Notably, PTEN loss, when combined with TP53/RB1 loss, is reportedly significantly enriched in resistant CRPC.^27^ In the absence of PTEN, wild‐type PTEN acts to inhibit basal cell proliferation, and the loss of PTEN leads to the expansion of stem (SCA+)/progenitor (BCL2+) cells.^28^ This expansion subsequently induces NEtD via the upregulation of SOX2.^29^ Similarly, in SCLC, PTEN loss enhances NE lineage plasticity initiated by TP53/RB1 loss.^30^ Moreover, the PI3K/AKT/mTOR pathway has been confirmed to participate in NEtD,^31^, ^32^, ^33^ with PTEN inhibition and loss of PTEN function activating this pathway. Overall, PTEN loss is intricately involved in the formation of the NE lineage, complementing the effects of TP53/RB1 inactivation.

### 2.3 MYC family alterations

The MYC family, consisting of MYC, MYCN, and MYCL, located on chromosomes 8, 2, and 1, respectively, represents an early‐discovered group of oncogenes.^34^ Generally, MYC, MYCN, and MYCL exhibit mutual exclusivity.^35^ In SCLC, the functions of MYC and MYCL have been extensively documented, revealing distinct roles in SCLC lineage states. Specifically, MYCL is essential for SCLC–NE differentiation driven by NOTCH inactivation, while MYC activates NOTCH signaling to induce cell fate transformation from NE to non‐NE.^36^, ^37^ Moreover, additional research has demonstrated that MYC induces the transition from SCLC–ASCL1 (SCLC with high NE genes) to SCLC–NEUROD1 (SCLC with moderate NE genes).^11^

Of note, in some other literatures, MYC was reported to drive NEtD in LUAD. It is well known that LUAD and SCLC both can derive from type II alveolar epithelial cells (AT II). In 2024, Gardner et al.^33^ developed a genetically engineered lung tumorigenesis mode to mimic the de novo SCLC and NEtD SCLC. The authors reported that EGFR expression induces LUAD, whereas EGFR loss initiates SCLC. Moreover, they found AT II cell and pulmonary NE cell exhibit differential sensitivity to MYC expression. MYC expression directly promotes pulmonary NE cell to develop to SCLC, while AT II cells need PTEN loss and MYC expression coexistence to give rise to NEtD SCLC.

Similarly, in NEPC, 40% of cases exhibit MYCN amplification, a phenomenon observed in only 5% of prostate adenocarcinomas.^1^ MYCN binds to EZH2, enhancing H3K27me3 methylation, allowing prostate cancer to escape AR dependence, ultimately promoting the evolution of NEPC.^38^, ^39^, ^40^ In addition, MYCN works in tandem with anaplastic lymphoma kinase to induce NEtD via the WNT/β‐catenin pathway.^41^ Recently, a novel mechanism involving MYCN in NEtD was unveiled. After ADT, pyruvate kinase L/R expression was upregulated, translocating into the nucleus to bind to MYCN, consequently upregulating reactive oxygen species modulator 1 and NE markers expression while altering mitochondrial function.^42^ Taken together, the MYC oncogene family exhibits mutual exclusivity in different NE carcinoma histological subtypes, playing a pivotal role in regulating cell fate and differentiation into distinct lineages.

### 2.4 Aurora kinase A amplification

Aurora kinase A (AURKA), a pivotal mitotic serine/threonine‐protein kinase and a critical cell cycle regulator, also plays a role in stabilizing MYCN through direct binding. In NEPC, AURKA is frequently amplified, emphasizing its significance in this context.^43^ Recently, CXCR7 has been identified as highly expressed in NEPC compared with primary prostate cancer (PCa) or CRPC. It exhibits a positive correlation with NE markers and has been validated to promote tumor cell growth in both NEPC cancer cells and NEPC patient‐derived xenograft (PDX) models.^44^ Mechanistically, CXCR7 recruits ARRB2, leading to endocytosis, where the complex is internalized into vesicles and transported along microtubules to the Golgi apparatus. Subsequently, it interacts with AURKA, enhancing AURKA T288 phosphorylation and activating its signaling. The involvement of microtubules in the transportation of the CXCR7/ARRB2 complex suggests that microtubule‐targeted drugs, such as paclitaxel, may exert a synergistic antitumor effect when combined with an AURKA inhibitor.

### 2.5 Cell division cycle 7 upregulation

Cell division cycle 7 (CDC7), a serine‐threonine kinase, is required to initiate DNA replication, and inhibition of CDC7 impairs cancer cell growth through S phase.^45^ CDC7 recently has been identified as an oncogenic driver in targeted therapy‐induced NEtD of lung cancer and prostate cancer, which is induced upon TP53 and RB1 inactivation.^46^ Mechanistically, TP53 and E2F1 both can bind to CDC7 gene promoter and inhibits or promotes its transcription, respectively. CDC7 inhibitor simurosertib suppresses NEtD by inducing MYC degradation in vivo and prolongs lung and prostate adenocarcinoma response to therapy. In sum, CDC7, as a therapeutic target, can control lineage plasticity and NEtD, and its upregulation confers to acquired resistance to targeted therapies in lung and prostate adenocarcinoma.

### 2.6 DNA damage response genes alteration

DNA damage response (DDR) genes play a crucial role in maintaining the integrity of genomic DNA, and disruptions in DDR function are a hallmark of cancer.^47^ In response to DNA damage stimuli, both DNA single‐strand breaks (SSBs) and double‐strand breaks (DSBs) rapidly accumulate, leading to the recruitment and PARPylation of PARP1. This process facilitates the recruitment of downstream homologous recombination repair factors, such as BRCA1/2.^48^ BRCA1 and BRCA2 are essential for regulating DSB repair, and mutations in these genes can result in oncogene activation and the loss of tumor suppressor genes.

#### 2.6.1 Poly(ADP‐ribose) polymerase 1

The DNA repair enzyme poly(ADP‐ribose) polymerase (PARP) family comprises 17 distinct proteins, with PARP1 being extensively validated in preclinical models and clinical trials.^49^ PARP1, a nuclear enzyme and a prominent sensor of both DSBs and SSBs, mediates PAPylation and plays a crucial role in the timely and accurate DNA damage repair. Its expression is notably elevated in SCLC and NEPC.^50^ The PARP inhibitor (PARPi), Olaparib, is currently being evaluated not only for its clinical efficacy in antitumor activity in ovarian and breast cancer but also in mCRPC and SCLC. Notably, small cell NE carcinomas with BRCA1/2 deletions exhibit extreme sensitivity to PARPi.^51^

Approximately 25% of patients with mCRPC harbor genomic alterations in DDR genes.^50^ MYCN transcriptionally activates PARP1, increasing the expression of downstream DDR genes such as BRCA1, RMI2, POLE, and TOPBP1. Notably, MYCN amplification enhances sensitivity to PARP inhibitors. Combining MYCN knockdown with PARP inhibitor (Olaparib) synergistically suppresses the development and maintenance of NEPC.^50^ Another study elucidated that targeting PARP suppresses the MYCN/CDK5/RB1/E2F1 signaling pathway, inhibiting NEtD.^52^ Additionally, DSBs can stimulate the cGAS–STING pathway, inducing PD‐L1 expression and creating an immunosuppressive microenvironment,^53^ indicating that the combination of PARPi and PD‐L1 inhibitor can be a potential treatment modality for SCLC.

#### 2.6.2 ATM and ATR

ATM and ATR are key kinases belonging to the phosphatidylinositol 3‐kinase‐related kinase family, responsible for coordinating cellular responses to DSBs and SSBs or replication stress, respectively.^54^ Thomas et al.^55^ reported that in SCLC, especially in cases with high NE–TFs such as ASCL1 and NEUROD1, there is a notable response to the ATR inhibitor (berzosertib), leading to durable regressions. In contrast, SCLC subtypes with POU2F3/YAP1 did not show a significant response, suggesting that NE tumors are more likely to be sensitive to ATR inhibition.^55^

ATM, upregulated by MYCN, has been reported to promote NEtD and metastasis. Mechanistically, MYCN associates with EZH2 to downregulate miR‐421, subsequently upregulating ATM expression. This upregulation further drives resistance to enzalutamide and the NEtD process in C4‐2 prostate cancer cells, and these effects can be countered by the ATM inhibitor Ku60019.^56^ Taken together, those NE tumors expressing high levels of DDR genes can benefit from DDR inhibitors such as berzosertib, which provides a rational targeted therapy for NE carcinoma.

### 2.7 Cancer‐related regulator of actin dynamics loss

Tumor suppressor cancer‐related regulator of actin dynamics (CRACD), known as KIAA1211, was found markedly mutated in SCLC in 2015, ranking the third following TP53 and RB1.^57^ Until 2024, the role of CRACD in lineage plasticity in LUAD was unveiled. Kim et al.^58^ revealed that CRACD serves as a gatekeeper restraining NE lineage plasticity via scRNA‐seq analysis of GEMM. CRACD KO induces NE markers upregulation in LUAD A549 cell line, as well as in Cracd KO mouse. However, the molecular mechanisms of how CRACD inactivation induces to NEtD remain unknown.

### 2.8 SMARCA4 overexpression

Switch sucrose nonfermenting (SWI/SNF)‐related, matrix‐associated, actin‐dependent regulator of chromatin, subfamily A, member 4 (SMARCA4), also known as BRG1, is a catalytic subunit of the SWI/SNF chromatin remodeling complex. In prostate cancer, SWI/SNF complexes contribute to the process of NEtD in CRPC.^59^ In addition, SMARCA4 previously reported frequent loss of function mutated in LUAD.^60^ Concepcion et al.^61^ reported that SMARCA4 deficiency induced LUAD dedifferentiation. Interestingly, SMARCA4 loss of function mutation is not common in SCLC (1.5%), but highly enriched in the non‐NE subtype of SCLC.^23^ Considering this phenomenon, Redin et al.^62^ found that SMARCA4 binds to ASCL1 and NEUROD1 to alter chromatin accessibility, enhancing NE signatures. Moreover, SMARCA4 also induces REST (NE phenotype suppressor) alternative splicing by SRRM4. Conversely, pharmacological inhibition of SMARCA4 with FHD‐286 promotes NE subtype transform to non‐NE subtype in SCLC, and as well as activating ERBB signaling, therefore, sensitizes to afatinib. In sum, SWI/SNF component SMARCA4, as a chromatin remodeler, exerts a role of gatekeeper, sustains the NE phenotype, and its loss of function reverses the process of NEtD by REST splicing.

## 3 EPIGENETIC ALTERATIONS

Aside from highly genomic instability, significant epigenetic abnormalities, including alterations in DNA methylation, histone modifications (methylation, acetylation, ubiquitination, and others), and dysregulation of noncoding RNAs (ncRNAs), play a direct role in lineage plasticity and NE reprogramming.^63^ These epigenetic alterations involve intricate interactions between DNA methylation and histone modifications, collectively regulating chromatin accessibility and driving aberrant transcription.^64^

### 3.1 DNA methylation

DNA methylation, an important epigenetic modification, plays critical roles in determining cell type and lineage via regulating genome stability and gene expression. The process of DNA methylation is mainly mediated by DNMT enzymes (DNMTs, referred to as “writers”), DNA demethylation enzymes (ten eleven translocations (TETs, referred to as “erasers”)), and methyl‐binding proteins (readers). Aberrant DNA methylation, a hallmark of cancer, typically occurs by transferring the methyl group onto 5‐carbon of the cytosine to form 5‐methylcytosine (5mC). 5mC in the gene promoter region can lead to the transcriptional silencing of tumor suppressor genes, contributing to tumorigenesis and progression.^65^

#### 3.1.1 DNA methyltransferases

DNA methylation is mainly catalyzed by DNMTs (consisting of DNMT1, DNMT3A, and DNMT3B), of which DNMT3A and DNMT3B are responsible for de novo DNA methylation while DNMT1 critical for maintenance of DNA methylation.^66^ In the context of NEPC, DNMT proteins are significantly upregulated compared with CRPC. Specifically, DNMT1 exhibits the highest upregulation, followed by DNMT3A in NEPC.^67^ Moreover, the pan‐DNMT inhibitor, decitabine, has been shown to attenuate cancer cell growth in PDX‐based NEPC models, GEMM, and NEPC or NEPC‐like cancer cell lines. However, DNMT inhibition could lead to the epigenetic upregulation of B7‐H3 (also known as CD276), a coinhibitory checkpoint. Consequently, combining decitabine with a B7‐H3 antibody–drug conjugate has shown the potential to exhibit an antitumor effect in NEPC.^67^ Taken together, DNMTs may play a role in promoting NE lineage progression in prostate cancer.

#### 3.1.2 Ten‐eleven translocation enzyme 2

Ten‐eleven translocation enzymes, including TET1, TET2, and TET3, drive the demethylation of DNA and oxidize the methyl group of 5mC into 5‐hydroxymethylcytosine (5hmC), 5‐formylcytosine, and 5‐carboxylcytosine.^68^ Among the three TET family members, TET2 is important for cell fate and lineage during carcinogenesis.^69^ Recently, Xu et al.^70^ reported that TET2‐driven 5hmC modifications is highly enriched in stem‐like and neuronal lineages, crucial for lineage plasticity and AR‐targeted therapy resistance. Mechanistically, TET2 expression is facilitated by ZNF397 loss and AR‐independent mCRPC, different from the its role in AR‐dependent hormone sensitive prostate cancer. TET2 inhibitor can restore drug sensitivity to AR‐targeted therapy. Overall, TET2 has a key role in driving NEtD, and pharmacological targeting to TET2 benefits for developing effective therapeutic strategies for patients with advanced PCa.

### 3.2 Histone modification

Histones consisting of H2A, H2B, H3, and H4 are highly conserved and constitutive proteins. The N‐terminal tails of these subunits are rich in lysine or arginine residues, making them susceptible to various epigenetic modifications.^71^ Histone modification, a posttranslational modification and key epigenetic regulatory mechanism in chromatin structure, accessibility, and the biological behaviors of malignancies, is a reversible covalent modification. The occurrence, removal, and effectiveness of this covalent modification are primarily regulated by histone‐modifying enzymes and their corresponding partners, which fall into three categories: writers (methyltransferases, acetyltransferases, etc.), erasers (demethylases, deacetylases, etc.), and readers.^72^ Alterations in histone modifications can change the expression pattern of the target genes. Increasing evidence suggests that epigenetic abnormalities in histone modifications drive lineage plasticity in NEtD tumors.

#### 3.2.1 Histone methylation

Histone methylation involves adding a methyl group (−CH_3_) to lysine or arginine residues in the tails of histone subunits and occurs in either a monomethyl (me), dimethyl (me2), or trimethyl state (me3).^73^ This modification, catalyzed by histone methyltransferases (HMTs, referred to as “writers”), can be reversed by histone demethylases (HDMs, referred to as “erasers”). In general, methylation at different loci of histones (H3K4, H3K9, H3K27, H3K36, and H4K20) has significant implications for the transcriptional regulation of genes. For example, methylation at H3K9me3 and H3K27me3 has been associated with transcriptional inhibition, while methylation at H3K4me1/2/3 and H3K36me3 has been associated with transcriptional activation.

*EZH2*. EZH2, a HMT at H3K27 site and the subunit of polycomb repressor complex 2 (PRC2), is widely acknowledged to be overexpressed in multiple types of cancer,^74^ mainly showing high expression in NE prostate cancer.^20^ The mechanisms of EZH2‐mediated epigenetic regulation contributing to NEtD have gradually been uncovered in recent years. In NEPC, EZH2 overexpression enhances the level of H3K27me3 and the expression of NE markers CHGA/B, while inhibitors of EZH2, such as GSK126 or Dznep, decrease the expression of NE markers. An upstream activator of EZH2, cAMP response element‐binding protein (CREB), was found to promote NE phenotypes following ADT. CREB is overexpressed and activated, with increased phosphorylation at the S133 site in NCI‐H1660 NEPC cells and enzalutamide‐treated prostate cancer cells, and this effect is reversed by dihydrotestosterone. Activators of CREB signaling, such as forskolin or isoproterenol, enhance EZH2 function, leading to H3K27me3 upregulation and downregulation of EZH2‐targeted genes DAB21P and ADRB2, as well as overexpression of the NE marker ENO2. Conversely, CREB signaling inhibitors like propranolol (PRO) and ICI reverse these effects.^75^

In SCLC, EZH2 has also been found to promote the NEtD process, and its inhibition facilitates the conversion to the non‐NE phenotype.^76^ Mechanistically, in NE–SCLC tumor cells, EZH2 silences the expression of TAP1, AXL, and YAP1 by elevating the levels of H3K27Me3 and reducing the levels of H3K27Ac.^76^ Notably, AXL and YAP1 are reported to be expressed in the non‐NE–SCLC subtype.^77^ Chromatin immunoprecipitation sequencing (ChIP‐seq) analysis revealed that the H3K27Ac peaks of major histocompatibility complex class I (MHC‐I)‐related genes are notably located at the TAP1 locus in non‐NE–SCLC cancer cells. This indicates that TAP1, when functioning properly without EZH2 inhibition, could potentially contribute to the recovery of MHC‐I expression.^76^ Taken together, EZH2 plays a critical role in lineage plasticity both in prostate cancer and SCLC–NEtD.

*EHMT2/G9a*. Euchromatic histone lysine methyltransferase 2 (EHMT2), also known as G9a, mediates mono‐ and demethylation of H3K9 residue.^78^ G9a has been observed overexpression in multiple cancer types and its inhibitors have been developed as potential agents. Recently, Yang et al. have identified G9a as an epigenetic driver in NSCLC–NEtD.^79^ Mechanistically, G9a increases the methylation of the SFRP1 promoter region and repress its expression, subsequently activated WNT/β‐catenin signaling and EGFR‐TKI‐induced NEtD. And, G9a inhibitor UNC0642, combined with erlotinib, not only significantly reverses SCLC transformation but also exerts synergistic antitumor effect in vivo. In sum, the role of G9a in driving NEtD enhances the understanding of histologic transformation in lung cancer and the mechanisms of resistance to EGFR‐TKIs,

*KMT2D*. Methyltransferase lysine methyltransferase 2D (KMT2D), also known as MLL2, is responsible for the mono‐methylation of H3K4 residue at gene enhancer sites. KMT2D exhibited loss of function mutations in 8% of SCLC tumors and 17% of SCLC cell lines^80^ also was one of top five mutated genes in high‐grade NE cervical cancer.^81^ KMT2D mutations are associated with global reductions in H3K4me; however, the detailed mechanism of KMT2D loss contributing to NE lineage development remains unclear.

#### 3.2.2 Histone demethylation

HDMs, responsible for demethylation on lysine or arginine residues, consists of histone lysine‐specific demethylase (LSD), arginine demethylase, and Jumonji C‐domain‐containing (JMJD) proteins family. JMJD family remove methyl both on lysine or arginine residues, contain KDM2, KDM3, KDM4, KDM5, KDM6, KDM7 family, and others.^82^

*KDM1A/LSD1*. Lysine‐specific demethylase 1 (KDM1A), also known as LSD1, is a HDM that specifically targets H3K4me1/2. It is highly upregulated in NEPC,^83^ SCLC,^84^ and Merkel cell carcinoma.^85^ The scaffold function of LSD1, rather than its catalytic function, is deemed more crucial for its growth‐promoting effects since NEPC cells are resistant to catalytic inhibitors.^86^ LSD1 was found to inactivate TP53 function by demethylating TP53 at K370.^87^ Inhibition of LSD1, either through RNA interference or with an allosteric LSD1 inhibitor (SP2509), has been shown to reactivate TP53 tumor suppressor pathways and reduce NEPC tumor growth.^83^ Additionally, LSD1, as a member of several repressive complexes, cooperates with histone deacetylases such as histone deacetylase 2 (HDAC2) to silence gene expression.^88^ In SCLC, inhibition of LSD1 by the selective inhibitor ORY‐1001 activates the NOTCH pathway, resulting in the upregulation of NOTCH1, NOTCH2, REST, and HES1. This leads to the inhibition of ASCL1 TF expression, consequently repressing NE reprogramming.^84^ Simultaneously, LSD1 inhibition increases the abundance of histone H3K27 acetylation along the NOTCH1 locus. The recent identification of a close correlation between LSD1 and MHC‐I epigenetic downregulation and an immunosuppressive microenvironment in SCLC is significant. In a multicenter, open‐label, phase I/II trial evaluating nivolumab alone or in combination with ipilimumab in SCLC patients, LSD1 expression emerged as a poor prognostic indicator for individuals treated with either nivolumab or the combination of nivolumab and ipilimumab.^89^ The inhibition of LSD1 with bomedemstat has been shown to restore MHC‐I expression and enhance T cell function, including increased IFN‐γ production. This effect further sensitizes the antitumor response to immune checkpoint blockade therapy in a GEMM harboring Rb1/Trp53 inactivation.^90^, ^91^ Nevertheless, the immunomodulatory role of LSD1 in NEPC has not been elucidated until now.

*KDM5A*. Lysine demethylase 5A (KDM5A), also known as RBP2 or JARID1A, functions as a demethylase targeting H3K4me1. In SCLC, KDM5A has been reported to repress the NOTCH (driving non‐NE) pathway, consequently promoting ASCL1 transcription, which is associated with the NE phenotype.^92^ Loss of function in KDM5A has the opposite effect, leading to increased ASCL1 expression and NE reprogramming. Moreover, combining a KDM5A inhibitor with an LSD1 inhibitor has been shown to synergistically repress ASCL1 expression in SCLC cancer cell lines. In prostate cancer, KDM5A is overexpressed and has been associated with a poor prognosis for patients. It facilitates tumor progression through the KDM5A/miRNA‐495/YTHDF2 axis.^93^ However, the role of KDM5A in NEtD of prostate cancer remains uncovered.

*KDM6A*. Lysine demethylase 6A (KDM6A), located on Chromosome X and also known as UTX, is a HDM that removes di‐ and trimethylation from lysine residues on H3K27. Intriguingly, KDM6A is involved in both demethylation and methylation processes. Beyond its role in demethylating H3K27me3, KDM6A also functions as a scaffold in the COMPASS complex (KDM6A and MML3/4), which methylates H3K4me1.^94^ The dual roles of KDM6A depend on its functional status; specifically, KDM6A gain of function tends to elevate H3K4me1 and deplete H3K27me3, while KDM6A loss of function mutations lead to decreased H3K4me1 and increased H3K27me3. Approximately 3−4% of SCLC patients harbor KDM6A mutations.^57^ To assess the distinct roles of KDM6A in the NEtD of SCLC, Duplaquet et al.^95^ investigated KDM6A inactivation mutations in an autochthonous SCLC mouse model. This resulted in the loss of chromatin accessibility at ASCL1 target genes binding enhancers, with a relative depletion at inflamed/interferon (IFN‐γ) genes. Meanwhile, there was increased accessibility at the NEUROD1 promoter.^95^ These findings align with the observation that the NE–SCLC subtype (SCLC–ASCL1) is poorly enriched in inflamed genes, while KDM6A loss promotes the transition of SCLC molecular subtype from ASCL1+ NE‐high to NEUROD1^+^ NE‐moderate, accompanied by an upregulation in inflammatory genes. In summary, KDM6A functions to promote ASCL1 transcription and drive the transition to the NE lineage, while the loss of KDM6A inversely induces a transition to a non‐NE molecular subtype.

*KDM7B*. KDM7B, also known as PHF8, is a histone lysine demethylase, belonging to JMJD family. PHF8 was reported highly expressed in NEPC and drive the development of NEPC. Additionally, PHF8 knockout blocked NE signature and tumor development in TRAMP mice. Mechanistically, PHF8 demethylates and removes repressive histone markers (H3K9me1/2, H3K27ME2, and H4K20me1) on the promoter region of the NE driving TF, FOXA2, resulting in upregulating FOXA2 expression and enhancing NE phenotype.^96^ Therefore, targeting PHF8 could be a promising therapeutic strategy for NEPC.

#### 3.2.3 Histone acetylation

Lysine residues also can be added with an acetyl group (−CH3CO) donated by acetyl‐CoA and catalyzed by histone acetyltransferase (HAT) “writer,” making chromatin relaxed and transcription activated and can be identified with acetyllysine “readers” (bromodomain and extraterminal family, BET), whereas also can be removed with HDAC “eraser,” making chromatin compacted and transcription repressed.

*CREBBP*. CREB binding protein (CREBBP), an HAT, is one of the most frequently mutated genes in SCLC (∼30%), contributing to the NE phenotype.^57^ CREBBP is known to function as a tumor suppressor in various cancers, including diffuse large B‐cell lymphoma^97^ and ESCC.^98^ However, it functions as an oncogene in prostate cancer.^99^ CREBBP inactivation, particularly in cooperation with Rb1/Trp53 loss, contributes to SCLC and is implicated in NE thyroid and pituitary carcinomas. Mechanistically, CREBBP inactivation reduces histone acetylation of cell adhesion genes such as CDH1.^100^ Interestingly, CREBBP expression alteration was not observed in enzalutamide‐resistant (Enz‐R) prostate cancer cells. However, the CREBBP inhibitor C646 sensitized Enz‐R prostate cancer cells to enzalutamide, downregulating ribosomal proteins RPL36 and RPL29.^101^ Taken together, CREBBP inactivation drives NE features in SCLC and enhances sensitivity to enzalutamide in resistant prostate cancer cells, but its role in NE lineage reprogramming remains unknown.

*HDAC*. Zhao et al.^102^ reported that a genome‐wide screen identified PAX9 overexpression associated with HDAC activity driving SCLC cell fate to differentiate into the NE lineage. PAX9 was found to bind directly to nucleosome remodeling deacetylase complexes such as HDAC1, acting as a transcriptional repressor. Pharmaceutical inhibition of HDAC using MERCK60 abrogated PAX9‐mediated transcriptional repression.

Recently, Oh et al.^103^ validated that HDAC inhibitor, fimepinostat, could restore EGFR expression in SNU‐2962A cells and their organoid model and augment the sensitivity of SCLC to EGFR‐TKI both in vitro and in vivo models. Overall, these findings suggest that HDACs participate in the progression of NEtD, indicating HDAC can be identified as a promising treatment strategy for NEtD of EGFR‐mutant LUAD.

*BRD4*. BRD4, a member of the BET protein family, sensing hyper‐acetylated histone tails through its bromodomains, regulates chromatin structure and gene expression, particularly in transcription elongation and super‐enhancer regulation.^104^ The role of acetylation reader protein in NEtD is rarely unveiled. Kim et al.^105^ revealed that NEtD in prostate cancer is dependent on E2F1/BRD4‐regualted program. Mechanistically, AR antagonist enzalutamide increases H3K27Ac level, and recognized by BRD4, then cooperate with NE‐lineage TF, E2F1, to promote NEtD and confer enzalutamide resistance. BETi, JQ1, blocks NE signatures expression and inhibits NEPC cell survival. The cooperativity between E2F1 and BRD4 indicates that the rational combination of their inhibitors can be applied for restrain NEtD of mCRPC.

#### 3.2.4 Histone ubiquitylation

TRIM59, identified as a putative E3 ubiquitin ligase, has been reported to be highly upregulated in NEPC and is implicated in promoting NEtD. In CRPC, the expression of TRIM59 is regulated by AR signaling. Specifically, in the presence of AR signaling, TRIM59 expression is repressed as AR binds to its promoter. However, in the absence of AR signaling due to androgen blockade, TRIM59 is derepressed. Subsequently, elevated TRIM59 levels enhance the ubiquitylation of P53 and RB1, further inducing the occurrence of NEtD.^106^

#### 3.2.5 Histone lactylation

Lactate, generated during the Warburg effect (aerobic glycolysis), has long been regarded as merely a byproduct of glycolysis, gradually recognized as a hallmark of cancer.^47^ In 2019, Zhang et al.^107^ first identified that histone lysine lactylation (Kla), a novel posttranslational modification, differs markedly from established acylation modifications through the unique addition of a lactyl group to a lysine residue, predominantly located on H3K18 and H3K4. Such nonmetabolic function of lactate on histone also alters chromatin structures and constrain gene expression. Histone lactylation, therefore, offers a valuable opportunity to enhance our comprehension of lactate's functions and its involvement in various cancer malignant behaviors including NEtD.

Wang et al.^108^ recently reported that ZEB1, an important epithelial–mesenchymal transition (EMT)‐associated TF, drives NE development of prostate cancer via histone lactylation‐mediated chromatin accessibility. Wang et al.^108^ demonstrated that ZEB1 positive in epithelioid cells are putative cells of origin for NEPC via lineage tracing on TRAMP mice. Of note, in TRAMP mouse model and scRNA‐seq analysis, ZEB1 was dynamically expressed during the process of NEPC. ATAC‐seq revealed that ZEB1 high expression exhibited more open chromatin structures than low expression counterpart. Specifically, ZEB1 transcriptionally regulates the expression of key glycolytic enzymes, including HK2, PFKP, and LDHA, thereby promoting glycolysis and lactate accumulation, further enhancing histone lactylation, resulting in NE plasticity.

Given the importance of mitochondria dysfunction in cellular lactate accumulation, in another study, He et al.^109^ demonstrated that NEPC and SCLC exhibit mitochondria dysfunction and aerobic glycolysis, and deficiency in the Numb/Parkin pathway drives NEtD in prostate or LUAD. Mechanistically, Numb, cell fate determinant, binds to and facilitates activation of the mitophagy initiator Parkin, and NUMB downregulation induces accumulation of depolarized mitochondria and metabolic reprogramming with lactate accumulation, subsequently leads to an upregulation of histone lactylation and then promotes the process of NEtD. In addition, restoration of Numb inhibits NEtD in prostate and LUAD.

In summary, histone lactylation, as the result of metabolic reprogramming in cancer cell, can facilitate NE lineage plasticity and promote targeted‐therapy resistance by remodeling the chromatin accessibility. However, the specific epigenetic writer, reader, and eraser for histone lactylation remain to be further investigated. Li et al.^110^ reported that E1A binding protein p300 (P300) and HDAC2 not only regulate histone acetylation, but also exert as the potential writer and eraser of histone lactylation in pancreatic ductal adenocarcinoma cells, respectively, indicating that acetylation and lactylation may competitively bind to the same histone lysine site and this competitive modification can exert complex regulatory effects on gene expression, influencing the process of NEtD.

### 3.3 ncRNA dysfunction

Beyond the coding gene, the ncRNAs, only 2% of the genome, regulate numerous biological behaviors, including lineage plasticity. Despite tremendous studies investigate the effects of ncRNAs in growth, invasion, migration, and other progressions in SCLC and CRPC, there are few studies deciphering the function of ncRNAs on NEtD. Here, we highlight the change and function of major components of ncRNAs as mediators in NE lineage transformation, such as long ncRNAs (lncRNA) and microRNAs (miRNAs).

#### 3.3.1 Long ncRNAs

lncRNA refers to ncRNA molecules with a length greater than 200 nucleotides that reportedly significantly regulate gene expression at the epigenetic level.^111^ There is an increasing consensus suggesting that dysregulation of lncRNAs contributes to tumor lineage plasticity. The aberrant expression of lncRNAs in the context of NEtD has been screened and well documented in studies utilizing preclinical models of NE prostate cancer, including lncRNA 19 and lncRNA‐p21.

H19, located on human chromosome 11p15.5, is predominantly active during fetal development and plays dual roles, functioning as both an oncogene and an antitumor factor in various biological processes, including genome instability and epithelial‐to‐mesenchymal transition.^112^ A recent temporal evolution analysis of a PDX‐based NEtD model investigated the dynamic landscape of lncRNAs during the NEtD process. Next‐generation sequence analysis identified H19, an epigenetic regulator, as highly expressed in NEPC.^113^ H19 is upregulated by SOX2 and binds to the PRC2, epigenetically modulating histone marks H3K27me3 and H3K4me3, which can reduce H3K27me3 level on NE genes and promote their transcription, and reduce H3K4me3 level on AR signature genes (AR, NKX3.1 and KLK2) and inhibit their expression. Concurrently, it prompts alterations in genome‐wide DNA methylation at CpG sites. The collective regulation of DNA and histone methylation further modulates NE expression.^114^

lncRNA‐p21, also known as TP53COR1, is a downstream repressor of p53 located on human chromosome 6p21.2.^115^ In comparison with adenocarcinoma, lncRNA‐p21 exhibits high expression levels in PDX‐based NEPC models, as well as in NEPC cancer cell lines such as NCI‐H660,^1^ NEPC‐like cells (DU145 and PC3), and enzalutamide‐resistant prostate cancer cells (C4‐2‐R, CWR22RV1‐R).^116^ lncRNA‐p21 reportedly drives NE differentiation by competing with HOTAIR for interaction with EZH2, ultimately enhancing STAT3 methylation.

#### 3.3.2 microRNAs

miRNA is a ncRNA with a small 21−22 nucleotides, binding to 3′ untranslated region (3′ UTR), and further posttranscriptionally represses mRNA expression. Dysregulation of miRNAs has been validated in promote or inhibit NE lineage transition. A small RNA next‐generation sequencing study identified downregulation of miR‐106a–363 cluster and upregulation of miR‐301a and miR‐375 were crucial for NEtD in NEPC, especially miR‐363 decrease contributed to NEtD by reduced binding to the 3′ UTR of AURKA and promoting its oncogenic expression.^117^

Let‐7 family, functioning as a tumor suppressor, was reported to inhibit NE lineage transition.^118^ Let‐7 is suppressed in NEPC DuNE cancer cell, and the panel of let‐7 targeted genes is upregulated. Mechanistically, let‐7 miRNA can be inhibited by stemness gene LIN28B, which subsequently results in HGMA2 and SOX2 downregulation and finally reduces NEPC tumorigenesis and tumor growth.

miR‐421 was found upregulated by MYCN overexpression and drove the development of NE prostate cancer.^56^ Mechanistically, MYCN promoted miR‐421 upregulation, consequently enhanced ATM expression and its phosphorylation, and then alleviated the senescence of castration‐resistant C4‐2 prostate cancer cell. Moreover, MYCN drove castration‐resistant C4‐2 cell invasion and migration by upregulating ATM via miR‐421.

miR‐194 expression in NEPC is elevated and inversely correlated with AR signaling.^119^ Mechanistically, HITS‐CLIP peak identifies that miR‐194 recognizes the FOXA1 3′ UTR and miR‐194 inhibits the FOXA1 expression level, whose loss of function drives NEtD. In addition, FOXA1 inactivation induced by miR‐194 is concomitant with IL‐8, Slug/ZEB1, and ERK signaling increase, which promotes tumor aggressiveness. In vivo study, miR‐194 inhibitor suppresses the growth of patient‐derived CRPC organoids with NE features.

Taken together, dysregulated lncRNAs and miRNAs potentially induce NE lineage switch, concomitant with other genomic or epigenetic alterations to drive NEtD. ncRNAs may serve as novel targeted therapeutic modality or molecular classifier for small cell NE carcinomas.

## 4 TFs ALTERATIONS

TFs are proteins whose expression is regulated by epigenetic modifications. They bind to specific DNA sequences in the downstream genes, activating or repressing their expression, thereby controlling diverse cellular processes and cell states.^120^ The comprehensive regulatory NE lineage master TFs is orchestrated by using single‐cell multiomics and ChIP‐seq. For example, master TFs such as ASCL1 and NEUROD1 have been identified as pioneering regulators determining cell fate and NE programming in NEPC and SCLC.^14^ Distinct TF expression during the development of NE transition allows the classification of SCLC and prostate cancer into different molecular subtypes (Figure 3).

### 4.1 ASCL1 and ASCL2 switch

Achaete‐scute homologue 1 (ASCL1), also known as ASH1, can be modulated by NOTCH signaling.^121^ Activation of the NOTCH pathway inhibits ASCL1 expression, leading to cell cycle arrest in SCLC.^122^ Conversely, downregulation of the NOTCH pathway increases ASLC1 transcription, promoting the NEtD process.

In NE prostate cancer, a novel molecular subtype of prostate cancer has been identified, consisting of class I (ASCL2+) and class II (ASCL1+) types. These subtypes share transcriptome similarities with SCLC–POU2F3 and SCLC–ASCL1, respectively.^15^ ASCL1 and ASCL2 are mutually exclusive during NEtD in prostate cancer, similar with the relationship between MYCL and MYC in SCLC and FOXA2 and FOXA1 in NEPC. Interestingly, ASCL1 and ASCL2 share a positive regulator, TFAP4, forming a reciprocal circuit. Mechanistically, TFAP4 binds to the promoter of ASCL1 and the enhancer of ASCL2. Knockout of TFAP4 was found to decrease ASCL1/2 expression, inhibiting cell growth in ASCL1+ prostate cancer cells but causing a drastic increase in ASCL2+ cells.^15^

Although ASCL1 and NEUROD1 act as pioneering TFs for the NE lineage in preclinical models, drugs specifically targeting these cell fate determinants have not yet been applied in clinical trials. However, the role of ASCL1 and NEUROD1 in NEtD can be disrupted by the standard second‐line therapy, lurbinectedin, a pan‐inhibitor of RNA‐Pol II for oncogenic transcription inhibition.^123^, ^124^ Mechanistically, lurbinectedin preferentially targets the CpG islands located downstream of the transcription start site, inducing ubiquitin/proteasome degradation of elongating RNA‐Pol II, leading to DNA damage response signaling (DSBs). Eventually, resistance to lurbinectedin can occur, indicating DDR inhibitor like ATR inhibitor combining with lurbinectedin may synergistically exert antitumor effects.

### 4.2 TF POU class 2 homeobox 3

TF POU class 2 homeobox 3 (POU2F3) is essential in driving the tuft (non‐NE) cell lineage in SCLC.^125^ The two coactivators of POU2F3, OCA‐T1 (C11orf53) and OCT‐T2 (COLCA2), are crucial for POU2F3 function by maintaining its chromatin accessibility and enhancer activity.^126^, ^127^ POU2F3 is exclusively expressed in the SCLC subtype without or with minimal NE markers, along with the expression of SOX9 and ASCL2,^125^ and is also enriched in cases with PTEN loss and MYC amplification.^128^ Interestingly, SCLC–POU2F3 (non‐NE) exhibits a more significant reduction in cell growth response to lurbinectedin in SCLC models than other subtypes,^129^ suggesting a better prognosis for this subtype. Previous studies have demonstrated that SCLC–POU2F3, accompanied by MYC amplification, predicts sensitivity to lurbinectedin.^129^

In prostate cancer, POU2F3 expression is observed in both CRPC and NEPC, and it appears to be mutually exclusive with ASCL1. However, POU2F3 expression is positively correlated with ENO2, another NE marker,^130^ contrary to the findings in non‐NE–SCLC. Taken together, these findings suggest that POU2F3 is more likely to serve as a master TF for NEtD in prostate cancer, while in SCLC, it is more pronounced as a non‐NE TF.

### 4.3 FOXA2 and FOXA1 switch

FOXA2/1, two members of the FOXA subfamily of forkhead box TFs, were first reported to be differentially expressed and have distinct effects on AR signaling in prostate cancer in 2005.^131^ In 2008, FOXA2 was found to be highly expressed in a mouse model with NE differentiation.^132^ In 2010, it was reported that FOXA2 expression, combined with HIF‐1α, is required for hypoxia‐mediated NE phenotype development.^133^ In 2022, based on a temporal evolution study using a GEMM with TP53/RB1/PTEN triple knockout, Han et al.^16^ deciphered the atlas of prostate cancer lineage plasticity using single‐cell ATAC and RNA sequencing. Prostate cancer was divided into five molecular patterns based on distinct TF expression, including ARPC–TFF3, ARPC–CLU, NEPC–MKI67, NEPC–ASCL1, and NEPC–NFIB. NEPC–ASCL1 and NEPC–NFIB were found to be spatially and terminally differentiated from the origin of ARPC–TFF3 and ARPC–CLU. During the late stage of NEtD (NEPC–ASCL1 and NEPC–NFIB), FOXA2, along with SOX2, another NE pioneering TF, exhibited prominent enrichment. In contrast, FOXA1 preferred to modulate the luminal lineage. Further ChIP‐seq analysis characterized FOXA2 as mainly directly binding to NE markers, the KIT locus, and activating the KIT pathway, while FOXA1 is specifically bound to the AR. Recently, Wang et al.^134^ demonstrated that FOXA2 collaborates with JUN and facilitates luminal epithelial transitions to multiple lineages. Taken together, FOXA2 and FOXA1 switch play critical roles in mediating NE lineage transition.

### 4.4 One cut domain family member 2

In 2018, ONECUT2 (one cut domain family member 2, OC2) was identified as a master TF in NE tumors in a pan‐NE cancer analysis.^135^ OC2 suppresses most AR‐targeted genes in castration‐resistant prostate cancer (CRPC). Furthermore, OC2 represses FOXA1 expression, mediating NEtD. OC2 expression was found to be higher in NE prostate cancer compared with CRPC tissues. Deletion of REST, a NE repressor, resulted in OC2 upregulation by binding to its promoter, and REST expression was inversely related to OC2 expression. Additionally, another study in 2019 showed that the function of OC2 in NEPC was closely correlated with HIF1α, which was regulated by OC2 binding to chromatin through SMAD3 activation.^136^ These studies collectively demonstrated that OC2 promotes NE phenotypic conversion in NEPC from different perspectives. However, the role of ONECUT2 in SCLC has not been established.

### 4.5 SOX2 and SOX9 switch

The stem‐like characteristics are one of the hallmarks of cancer, as cancer cells often exhibit features similar with stem/progenitor cells. Increasing evidence indicates that the SRY‐box (SOX) family of TFs dynamically regulates lineage plasticity, with examples such as SOX2 and SOX9. NEtD shows high stem‐like features, characterized by elevated expression of SOX2, a gene associated with cancer stem cells. The loss of TP53/RB1 promotes NE‐lineage transition by increasing the expression of SOX2 and EZH2. Conversely, the restoration of TP53/RB1 and inhibition of SOX2 can reverse the NEtD process in prostate cancer.^19^, ^20^ Treatment with selinexor, an exportin 1 (XPO1) inhibitor, leads to downregulation of SOX2 and NE dedifferentiation in both NEPC and SCLC.^31^ In gastrointestinal NE carcinoma, SOX2 promotes NEtD under the hypermethylation of ASCL1's promoter region.^137^ Thus, SOX2 expression is upregulated in various types of NE carcinoma, including SCLC, NEPC, and gastrointestinal NE carcinoma.

SOX9, another crucial factor in stem/progenitor cell development, contributes to tumor progression. The expression of SOX2 and SOX9 is mutually exclusive and is regulated under epigenetic control, influencing tumor growth and invasion in lung cancer.^138^ In lung cancer, SOX2 interacts with HDAC1 to inhibit SOX9 expression, while inhibition of HDAC1 increases SOX9 expression. In breast cancer, SOX9 is known to maintain breast luminal progenitor cells, and its expression can be reduced by SOX2.^139^ In addition, it was found that ASCL1 inhibits SOX9 expression. SOX9 expression, along with RUNX1 and RUNX2, was highly enriched in the non‐SCLC–ASCL1 subtype and human SCLC cell lines with low ASCL1 expression.^140^ Upregulation of SOX9 with ASCL1 loss leads to bone or cartilage differentiation, resembling an osteosarcoma‐like fate. The non‐SCLC–ASCL1 subtype was also enriched in HIPPO/YAP1 and NOTCH/REST signaling. A study in liver cancer also demonstrated that YAP and SOX9 play a similar role in determining hepatocyte plasticity, transitioning from mature hepatocytes to progenitor cells,^141^ consistent with YAP and SOX9 being both enriched in the non‐NE subtype. In summary, these studies indicate that two stem cell regulators, SOX2 and SOX9, govern different lineage development. Specifically, SOX9 promotes tumor cells to non‐NE differentiation, such as bone or cartilage differentiation, while SOX2 promotes the determination of the NE lineage. Additionally, SOX2 enhances NE‐specific ASCL1 transcription, reducing SOX9 expression.

### 4.6 Signal transducer and activator of transcription

In recent years, emerging evidence has shown that the JAK–signal transducer and activator of transcription (STAT) signaling pathway, known for its role in inflammatory response, is involved in NE lineage plasticity in prostate cancer, presenting significant therapeutic potential.^24^, ^142^ In this respect, Chan et al.^24^ indicated that JAK–STAT and FGFR pathways are activated following TP53/RB1 loss at the early time point and are accelerated by androgen blockade. Conversely, coinhibition of JAK and FGFR promotes the restoration of luminal lineage.^24^ Deng et al.^142^ also demonstrated that ectopic JAK–STAT activation enables the transition to a multilineage and stem‐like state, which can differentiate into various lineages, including the NE lineage, leading to resistance to androgen blockade. Another study consistently showed that the JAK–STAT1/IFIT5/SOX2 axis facilitates the acquisition of stemness in prostate cancer cells.^143^ Taken together, JAK–STAT signaling is implicated in driving lineage plasticity and acquiring resistance to targeted therapies in prostate cancer.

### 4.7 RE1‐silencing TF

RE1‐silencing TF (REST), also known as neuron‐restrictive silencer factor (NRSF), plays a crucial role in inhibiting NEtD in both SCLC and NEPC.^144^, ^145^ REST expression can be regulated by MYC or RUNX2 in a NOTCH‐independent manner,^11^ highlighting its involvement in non‐NE phenotypes that persist in the absence of NOTCH signaling and are driven by a RUNX2/REST‐dependent signaling pathway.^146^ Additionally, REST can drive the conversion of NE to non‐NE phenotypes in a NOTCH‐dependent manner, serving as a key component in determining non‐NE fate. The crosstalk and bidirectional regulation between the Hippo/YAP/TEAD and NOTCH/REST pathways potentially contribute to the transition from NE to non‐NE fate.^144^

### 4.8 Paired box 6

Paired box 6 (PAX6), a DNA‐binding TF, determines the human neuroectoderm cell fate.^147^ Recently, PAX6 has been recognized as a novel neural TF driving NEtD in prostate cancer.^148^ Specifically, PAX6 expression was notably upregulated in NEPC and inhibited by AR activation, whereas PAX6 upregulation drives NE phenotypes via enhancing MET/STAT5A‐mediated chromatin accessibility. Therefore, PAX6 might serve as a potential therapeutic target for the treatment of NEPC.

## 5 OTHER PROTEOMIC ALTERATIONS

### 5.1 Delta‐like protein 3

Delta‐like protein 3 (DLL3) is an inhibitory NOTCH ligand specifically expressed on the surface of NE carcinoma, including SCLC, NEPC, and small cell bladder cancer, making it a potential therapeutic target.^12^, ^149^, ^150^ DLL3 expression is regulated by NE–TF ASCL1 and correlated with NE phenotype.^12^

In prostate cancer, DLL3 overexpression was observed in 76.6% of NEPC cases, 12.5% in CRPC with adenocarcinoma features (CRPC‐adeno), and was not detectable in PCa.^151^ DLL3 expression is regulated by the NE‐specific TF ASCL1 and is correlated with the NE phenotype. While DLL3 inhibition with SC16LD6.5 markedly inhibited tumor growth in NEPC cell lines and PDX models, DLL3 was not identified as a master regulator for NEtD, as its inhibition did not consistently decrease NE markers. DLL3 overexpression on tumor cells is considered a potential target for NEPC treatment.

### 5.2 Tumor‐associated calcium signal transducer 2

Tumor‐associated calcium signal transducer 2 (TROP2), a surface glycoprotein, is overexpressed in various cancers, including NEPC, breast cancer, and NSCLC.^152^ TROP2 overexpression has been reported to drive NE reprogramming by accumulating DNA damage, as evidenced by increased phospho‐γH2A.X (double‐strand break markers) and activating PARP1. Consequently, TROP2‐overexpressing tumors are sensitive to PARP1 inhibitor Olaparib.^152^ The features of TROP2 overexpression on the surface of tumor cells and vulnerability to PARP inhibitors suggest a combination therapy strategy using anti‐TROP2 antibody–drug conjugates (ADC) and PARP inhibitors. In 2017, the anti‐TROP2 ADC sacituzumab govitecan, which carries SN‐38 (a topoisomerase I inhibitor similar with topotecan, a second‐line treatment), showed promising therapeutic effects in advanced SCLC.^153^ However, the role of TROP2 in NEtD in SCLC is not fully understood.

### 5.3 Adenosine receptor A2A

Adenosine receptor A2A (ADORA2A), a surface G protein‐coupled receptor for adenosine, primarily has been studied in the neuronal development. ADORA2A was recently reported selectively upregulated in NEPC and SCLC, and its signaling was critical for NE differentiation.^154^ ADORA2A deficiency in GEMM in prostate and lung cancer could ablate NEtD process, and pharmacological inhibition blockade with ADORA2A antagonist SCH58261 also exhibited a promising antitumor effect in both NEPC and SCLC. Mechanistically, ADORA2A activation drives proline synthesis via ERK/MYC/PYCR1/2 axis and then enhances SIRT6 and SIRT7‐mediated H3 deacetylation, resulting in H3K27ac downregulation and confer NE transition. Thus, membrane protein ADORA2A upregulation and activation reveal a new mechanism of NEtD in NEPC and SCLC via ERK/MYC/PYCR/SIRT6/7 axis and is a potential druggable target in NE carcinoma.

### 5.4 Chlorogenic receptor muscarinic 4

Chlorogenic receptor muscarinic 4 (CHRM4), a surface G protein‐coupled receptor for acetylcholine, is known to play critical role in cholinergic signals transduction in central nervous system. CHRM4 was reported correlated with ADT resistance and NEPC development.^155^ Mechanistically, ADT induces CHRM4 expression upregulation, and AR activated by androgen can conversely repress CHRM4 expression. CHRM4 activates AKT/MYCN signaling to upregulate interferon alpha 17 (IFNA17), then drives NE marker and immunosuppressive checkpoints overexpression (CTLA4 and PDL1).^156^ Additionally, pharmacological inhibition of CHRM4 with certinib suppresses tumor growth and NEtD of NEPC. In summary, ADT‐induced CHRM4 overexpression and activation drives NEtD and immunosuppressive tumor environment through AKT/MYCN/IFNA17 axis, which confers targeting CHRM4 as a potential treatment for NEPC.

### 5.5 Mucin1‐C

Mucin 1 (MUC1), a transmembrane heterodimeric protein, consists of two subunits, the N‐terminal subunit and the C‐terminal subunit (MUC1‐C). MUC1‐C is involved in lineage plasticity for NEtD in prostate cancer, with its expression correlating with POU3F2 (BRN2) and the NEPC score,^157^ of which BRN2 has been identified as NE master TF in NEtD.^158^ MUC1‐C interacts with MYCN to bind to the promoter of POU3F2, activating its expression and enhancing SOX2 to drive NEtD. Therefore, targeting MUC1‐C could be a potential therapeutic strategy for attenuating NEPC progression.

### 5.6 Fibroblast growth factor receptor 1

Fibroblast growth factor receptor 1 (FGFR1), a membrane protein known as an NSCLC‐type alteration, is frequently amplified in SCLC.^35^ In 2020 and 2022, FGFR1 was found to promote NEtD in both SCLC and NEPC, respectively.^24^, ^159^ Analysis of ligand–receptor pairs in an NEPC GEMM model prioritized FGF1–FGFR as a top candidate for lineage plasticity. Inhibition of FGFR restored luminal lineage identity with increased CK8 and AR expression and decreased mesenchymal lineage marker VIM. Recently, another study also reported that FGF, FGFR ligand, as an inducer of the MAPK signaling pathway, promote the growth and metastasis of SCLC and facilitate the transformation of LUAD into SCLC.^33^ Overall, FGFR1 serves as a novel NEtD driver with great therapeutic target potential.

### 5.7 XPO1

XPO1, a nuclear exporter and secreted protein, has been approved by the United States Food and Drug Administration (US FDA) for multiple myeloma.^160^ XPO1 is overexpressed in SCLC and NEPC. Mechanistically, the loss of TP53/RB1 induces XPO1 overexpression, promoting NEtD via the upregulation of SOX2.^31^ Inhibition of XPO1 by selinexor sensitizes NEPC and SCLC to chemotherapy and interferes with NEtD by downregulating ONECUT2 and SOX2 in both lung and prostate models. The XPO1/SOX2 pathway participates in lineage plasticity to promote NEtD in NEPC and SCLC.

### 5.8 Ubiquitin carboxy‐terminal hydrolase L1

Ubiquitin carboxy‐terminal hydrolase L1 (UCHL1), a secreted protein, is a deubiquitinating enzyme regulating protein stability. UCHL1 is notably expressed in NEPC and SCLC, promotes cell growth in NEPC (NCI‐H660), NE‐like (DU145), and SCLC (NCI‐H82) cancer cell line, and upregulates NE signatures in CRPC‐adeno (22RV1) cell line in vitro.^161^ Consistently, UCHL1 overexpression drives NE markers in CRPC‐adeno tumor xenograft models in vivo. In addition, UCHL1 overexpression promotes cancer cell migration and metastasis in NEPC and SCLC. Mechanistically, UCHL1 destabilizes p53 and stabilizes POM121 by regulating their ubiquitination, and then promotes nucleoporin POM121‐mediated nuclear localization of two oncogenic TFs, c‐MYC and E2F1, resulting in NE differentiation. In addition, UCHL1‐specific inhibitor, LDN‐57444, significantly reduced NE markers expression, as well as delayed tumor growth of multiple NE carcinoma xenografts, including NEPC (NCI‐H660), SCLC (NCI‐H82), and neuroblastoma (IMR‐32) cell line, and NEPC and SCLC PDX models in vivo.^161^ Overall, UCHL1 is identified as a therapeutic target in NEPC and SCLC.

## 6 EMERGING THERAPEUTIC TARGETS AND CLINICAL TREATMENT STRATEGIES

Due to significant epigenetic alterations in NEtD with considerable therapeutic target potential, numerous clinical trials have been completed or are under evaluation, focusing on epigenetic inhibitors such as DNMT, EZH2, PARP, ATR, and LSD1 inhibitors. Our summary primarily focuses on ongoing or completed clinical trials related to epigenetic therapy for SCLC, NEPC, and other NE carcinomas with reported or published efficacy. Additionally, we provide a general overview of trials with recruiting or active but not yet recruiting status in Table 1. Detailed information about these trials can be accessed on ClinicalTrials.gov.

**TABLE 1:** The clinical trials of acquiring vulnerabilities in neuroendocrine carcinoma.

Tumor	Target	Trail number	Phase	Patients	Interventions	Status
CRPC (NEPC)						
	AURKA	NCT01799278	II	31	Alisertib	Completed
	AURKA	NCT01094288	I	41	Alisertib + docetaxel	Completed
	PARP	NCT03834519	III	793	Olaparib + pembrolizumab vs. enzalutamide or abiraterone	Active, not recruiting
	PARP	NCT02987543	III	387	Olaparib vs. enzalutamide or abiraterone	Completed
	PARP	NCT02975934	III	405	Rucaparib vs. abiraterone or enzalutamide or docetaxel	Active, not recruiting
	PARP	NCT02952534	II	277	Rucaparib	Completed
	ATR PARP	NCT03787680	II	49	Olaparib + ceralasertib	Active, not recruiting
	HADC	NCT00878436	I/II	52	Panobinostat + bicalutamide	Completed
SCLC						
	AURKA	NCT02038647	II	178	Alisertib + paclitaxel	Completed
	AURKA	NCT01677559	I	31	Alisertib + paclitaxel	Completed
	PARP	NCT02734004	I/II	40	Olaparib + durvalumab	Active, not recruiting
	PARP	NCT02446704	I/II	50	Olaparib + temozolomide	Active, not recruiting
	PARP	NCT02484404	II	20	Olaparib + durvalumab	Recruiting
	ATR	NCT02487095	II	26	Berzosertib + topotecan	Active, not recruiting
	ATR	NCT04802174	I/II	75	Berzosertib + lurbinectedin	Recruiting
	ATR	NCT04514497	I	96	Elimusertib + irinotecan or topotecan	Active, not recruiting
	ATR	NCT05941897	II	38	Ceralasertib + durvalumab	Recruiting
	TROP2 ATR	NCT04826341	I/II	85	Sacituzumab govitecan + berzosertib	Active, not recruiting
	LSD1	NCT02034123	I	29	GSK2879552	Terminated
	LSD1	NCT05191797	I/II	34	Bomedemstat	Recruiting
	LSD1	NCT04350463	II	92	CC‐9001 + nivolumab	Active, not recruiting
	HADC	NCT01222936	II	21	Panobinostat (LBH589)	Completed
	HADC	NCT00958022	I	7	LBH589 + carboplatin, etoposide	Terminated
	HADC	NCT00697476	I/II	2	Vorinostat + topotecan	Terminated
	HADC	NCT00702962	I	8	Vorinostat + carboplatin, etoposide	Terminated
	DLL3	NCT03319940	I	107	Tarlatamab	Recruiting
	DLL3	NCT01901653	I	82	Rova‐T	Completed
	DLL3	NCT03086239	I	29	Rova‐T	Completed
	DLL3	NCT02674568	II	339	Rova‐T	Completed
	DLL3	NCT03061812	III	296/148	Rova‐T vs. topotecan	Completed
	DLL3	NCT03026166	I/II	42	Rova‐T + nivolumab plus or minus Ipilimumab	Terminated
	DLL3	NCT03033511	III	478	Rova‐T	Terminated
	XPO1	NCT02215161	II	14	Selinexor	Terminated
CRPC SCLC	EZH2	NCT03460977	I	267	PF‐06821497	Recruiting
CRPC Others	EZH2	NCT04388852	I	80	Valemetostat (DS3201)	Recruiting
NEPC SCLC BSCNC	ATR	NCT03896503	II	98	Berzosertib + topotecan	Active, not recruiting
NEPC SCLC Other NC	LSD1 HADC6	NCT05268666	II	126	JBI‐802	Recruiting
SCLC Other NC	PARP	NCT04701307	II	48	Niraparib + dostarlimab	Active, not recruiting
SCLC Other NC	HADC	NCT00926640	I	28	Belinostat + carboplatin, etoposide	Completed
NEPC SCLC GEPNET	DLL3 CD47	NCT05652686	I	58	PT217	Recruiting

Abbreviations: CRPC, castration‐resistant prostate cancer; NEPC, neuroendocrine prostate cancer; SCLC, small cell lung cancer; BSCNC, bladder small cell neuroendocrine carcinoma; NC, neuroendocrine carcinoma; GEPNET, gastroenteropancreatic neuroendocrine tumors.

### 6.1 Targeting AURKA amplification

In a phase II trial of the AURKA inhibitor alisertib, NEPC patients with AURKA and MYCN activation experienced significant clinical benefit despite the study not meeting its primary endpoint.^162^ A phase II trial of alisertib with paclitaxel demonstrated improved progression‐free survival (PFS) in refractory SCLC cases with MYC amplification.^163^ Another phase I study in 2021 also indicated that alisertib with paclitaxel has a manageable side‐effect profile and promising preliminary efficacy in high‐grade SCLC.^164^ Therefore, inhibition of AURKA will be an attractive therapeutic modality for NE carcinoma.

### 6.2 Targeting DNA damage repair signaling

#### 6.2.1 | Targeting PARPi

Targeting DNA damage repair with PARP inhibitors has emerged as a potential therapeutic strategy and has been extensively investigated in clinical trials for relapsed SCLC and mCRPC. While PARPi alone has shown moderate antitumor activity in SCLC, combination treatment strategies involving PD‐L1 inhibitors or temozolomide have been explored. However, a phase I/II trial combining the PARPi olaparib with durvalumab (a PD‐L1 monoclonal antibody) demonstrated only 28.9% of patients achieving disease control at week 12, dropping to 5.3% at week 28, with an objective response rate of 10.5%.^165^ Another phase II trial of olaparib plus durvalumab reported modest efficacy, at least in this 20‐patient cohort.^166^ Additionally, a combination strategy in a phase I/II trial of olaparib plus temozolomide showed promising antitumor efficacy with an objective response rate of 41.7%, median PFS of 4.2 months, and median overall survival of 8.5 months.^167^

In mCRPC, olaparib has advanced to phase III trials and has indicated improved outcomes, particularly in patients with BRCA1/2 or ATM alterations, compared with AR inhibitors (abiraterone or enzalutamide).^168^ However, in a large mCRPC cohort of 793 patients, a phase III study of olaparib plus pembrolizumab (anti‐PD‐1 antibody) showed no significant improvement in PFA and overall survival compared with abiraterone or enzalutamide.^169^ Notably, CRPC with BRCA mutations has been shown to enhance sensitivity to PARPi, as demonstrated in the final results from the phase II TRITON2 study of rucaparib, where half of the enrolled patients with BRCA‐mutated metastatic CRPC experienced a complete or partial tumor size reduction with rucaparib.^170^

#### 6.2.2 | Targeting ATR

Due to frequent ATR alterations in NE carcinoma with significant therapeutic potential, the ATR inhibitor berzosertib, in combination with topotecan, has been extensively studied in SCLC. A phase II trial of berzosertib plus topotecan demonstrated a significant improvement in overall survival (8.9 vs. 5.4 months) but not in PFS (3.9 vs. 3.0 months) compared with topotecan alone.^171^ Another phase II trial of berzosertib plus topotecan also showed that berzosertib enhances the efficacy of topotecan in relapsed SCLC.^55^ Additionally, another ATR inhibitor, ceralasertib (AZD6738), in combination with olaparib for CRPC, is under evaluation in a clinical trial (NCT03787680).

### 6.3 Targeting LSD1

A phase I trial of the LSD1 inhibitor GSK2879552 showed poor disease control and a high incidence of adverse effects in SCLC, leading to its termination due to a low risk‐benefit profile.^172^ New trials of LSD1 inhibitors in SCLC, including JB1‐802 (NCT05268666) and bomedemstat (NCT05191797), are currently recruiting. Additionally, a phase II study of CC‐9001 (NCT04350463) is active but not recruiting and is focused onNE carcinoma, including SCLC, NEPC, and other NE‐derived cancers.^173^

### 6.4 Targeting HDAC

A phase I/II trial of the HDAC inhibitor LBH589 (panobinostat) in combination with bicalutamide (a second‐generation antiandrogen) demonstrated that HDAC inhibition reduces AR‐mediated resistance to bicalutamide in CRPC with tolerable toxicity.^174^ However, several trials testing HDACi in SCLC have been terminated, given its unsatisfactory antitumor profile.

### 6.5 Targeting surface marker DLL3

Considering the specific expression pattern of DLL3 on the surface of NE carcinoma, not on normal tissues, various therapeutic strategies targeting DLL3 have been developed to treat NE carcinoma, particularly in SCLC. These strategies include DLL3‐antibody–drug conjugates, DLL3‐targeted bispecific T‐cell engagers, and DLL3‐targeted chimeric antigen receptor T‐cell therapy (CAR‐T). Additionally, an ongoing phase I study of PT217 (dual antibodies to DLL3 and CD47, NCT05652686) in patients with advanced refractory SCLC, NEPC, and gastroenteropancreatic NE tumors expressing DLL3 is currently underway.

A humanized anti‐DLL3 ADC, employing a DNA‐damaging pyrrolobenzodiazepine (PBD) dimer toxin, induced durable tumor regression in multiple SCLC PDX preclinical models.^175^ Further, a phase I clinical study of the anti‐DLL3 ADC, rovalpituzumab tesirine (SC16LD6.5, also known as Rova‐T), demonstrated promising durable responses with a manageable safety profile.^176^ Another phase I study of Rova‐T also reported similar observations in the Japanese population,^177^ indicating Rova‐T could exert antitumor in multiple ethnic cohorts. Following a phase II single‐center clinical trial of Rova‐T involving 339 patients, modest clinical activity was observed irrespective of DLL3 expression levels in recurrent SCLC.^178^ The objective response rates for all patients, DLL3‐high, and DLL3‐positive patients were 12.4, 14.3, and 13.2%, respectively. However, no median overall survival benefits were observed. In the subsequent phase III study of Rova‐T versus topotecan (a standard second‐line treatment for SCLC), Rova‐T displayed inferior outcomes in overall survival with a higher incidence of adverse events than topotecan.^179^ A combination study of Rova‐T with nivolumab plus or minus ipilimumab in a phase I/II trial exhibited intolerable adverse effects alongside promising antitumor activity.^180^ When administered as a maintenance therapy following first‐line chemotherapy, Rova‐T showed no benefit in overall survival but demonstrated toxicity.^181^

Bispecific T‐cell engagers involving dual antibodies targeting two molecules have emerged as a clinically validated therapeutic approach in solid tumors. AMG 757, featuring anti‐DLL3 and anti‐CD3 antibodies, demonstrated a notable antitumor effect in preclinical models, particularly PDX of SCLC.^182^ Moreover, a phase I study of the bispecific T‐cell engager tarlatamab revealed a manageable safety profile along with durable antitumor activity in recurrent SCLC.^183^

In another study exploring DLL3‐targeting CAR T cell therapy, interleukin‐18 (IL‐18) was utilized to enhance the activation of both CAR T cells and endogenous T cells.^184^ Mechanistically, IL‐18‐secreting CAR T cells reprogrammed the myeloid compartment infiltration and induced an anti‐inflammatory/pro‐tumor phenotype.

Taken together, Rova‐T based on DLL‐3 ADC drug cannot prolong overall survival of patients with SCLC, whether administrated in first‐line follow‐up treatment or compared with topotecan second‐line treatment. And bispecific T‐cell engager and CAR T cell therapy need to be further validated in clinical trials.

## 7 CONCLUSION AND PROSPECTS

Given the high metastasis, recalcitrant behavior, and limited treatment options for NEtD tumors, understanding the underlying mechanisms and identifying potential therapeutic targets is crucial for improving clinical outcomes. Lineage plasticity is a major mechanism of resistance to targeted therapies. With molecular targeted drug intervention, a selective pressure, lineage plasticity involves the dedifferentiation of the mature epithelial (luminal) lineage into a multilineage state, allowing cancer cells to acquire stem/progenitor characteristics and further differentiate into the NE lineage,^143^ leading to acquired drug resistance. Of note, the key genomic alterations in NEtD tumors and de novo NE tumors exhibit high similarity, including TP53 and RB1, but also embrace distinct molecular alterations.^185^

**FIGURE 6:**
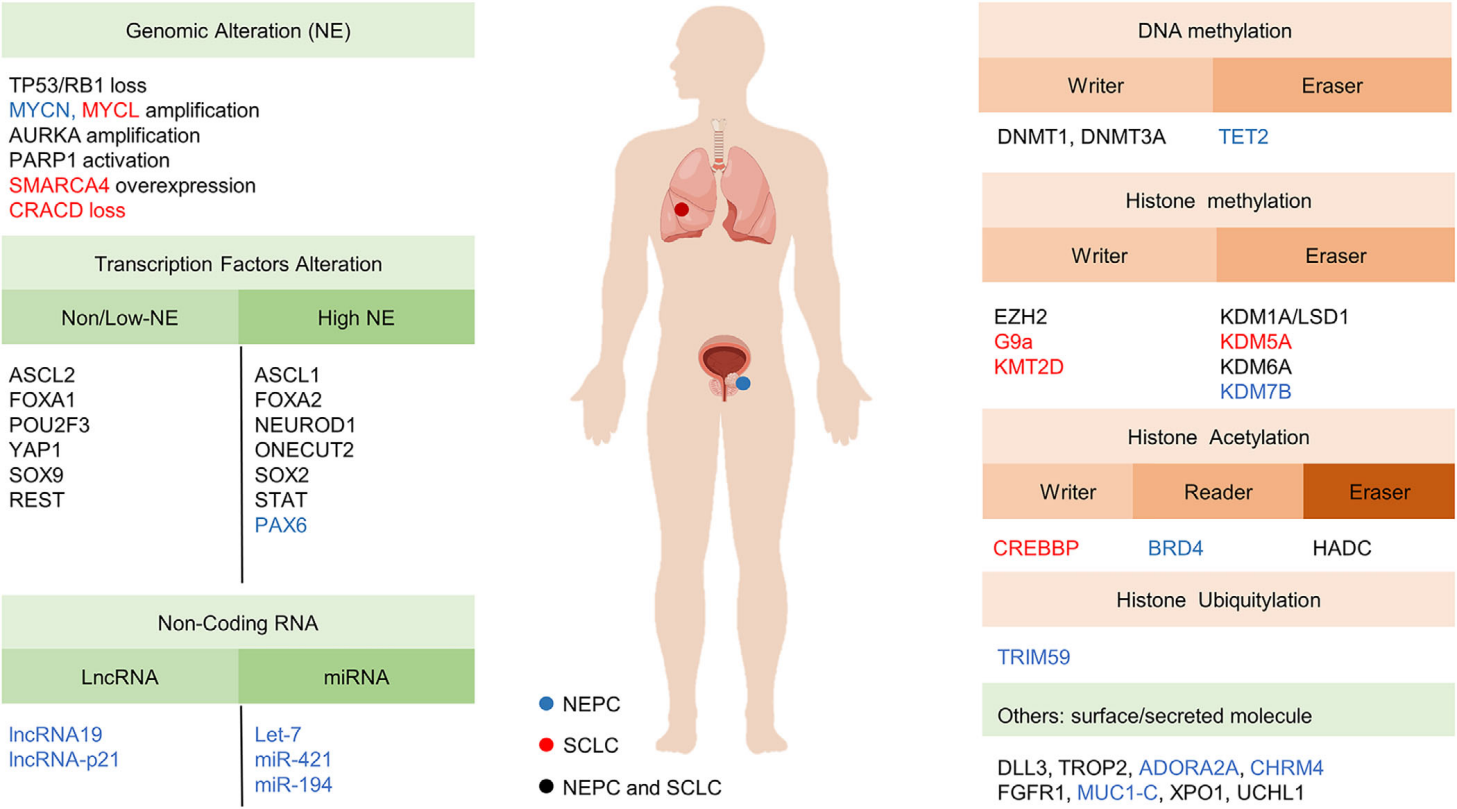
Molecular mechanisms and therapeutic targets in neuroendocrine transformation, including genomic alteration, epigenic regulations, dysregulated transcription factors, and surface or secreted protein aberrations.

Studies employing temporal evolution, lineage tracing, and spatial profiling on preclinical models, including GEMM, PDX, cell‐derived xenografts, and organoids, as well as clinical samples with single‐cell omics sequencing, have provided crucial insights into the dynamic evolution of molecular events in tumor progression. Interestingly, NEtD tumors in different tissues share convergent molecular subtypes and therapeutic vulnerabilities, albeit with some organ‐specific differences (Figure 6). For instance, both NEPC and SCLC share common genetic alterations (e.g., TP53/RB1/PTEN loss, MYC amplification), expression of NE master TFs (e.g., ASCL1, NEUROD1, FOXA2, SOX2), epigenetic alterations (e.g., DNMT, EZH2), the surface markers (e.g., DLL3, TROP2, FGFR), secreted protein (UCHL1), and nuclear exporter (XPO1) overexpression. In addition to pro‐NEtD genetic alterations, there are also genetic alterations that conversely inhibit NEtD and maintain an adenocarcinoma lineage state, such as TMPRSS2–ERG fusion inhibiting NEtD in prostate cancer^186^ and SMARCA4 loss reverse NEtD in SCLC.^62^

In addition, targeted‐induced NEtD occurs not only frequently in prostate cancer and SCLC, but also emerges in other cancers, such as small cell NE bladder cancers (SCBC). Similar with SCLC and NEPC, SCBC also exhibits frequent TP53/RB1 mutations, and distinct molecular subtypes characterized by unique lineage‐specific TFs, including ASCL1, NEUROD1, and POU2F3.^187^ Despite sharing convergent genetic mutations, SCBC also exhibits distinct mutation patterns. The bladder‐specific mutation in the TERT promoter was identified in nearly 95% SCBC patients, which is absent in SCLC.^25^, ^188^

The shared vulnerabilities could be exploited for the development of drugs targeting NEtD tumors. Throughout the development in the clinical application of epigenetic inhibitors in SCLC and NEPC, we can conclude that DNA damage response inhibitors of PARP and ATR inhibitors have shown promising antitumor activity. However, EZH2, which has been widely validated to drive NE lineage programming in preclinical models, has not still been approved for NE tumors treatment. The early clinical application of HDAC inhibitors in SCLC also have been terminated due to unsatisfactory efficacy and intolerable toxicity events.

The unsatisfactory or contradictory therapeutic effects of some epigenetic inhibitors are attributed to the dynamically evolving molecular subtypes within tumors. For instance, SCLC can be classified into four distinct molecular subtypes based on lineage‐defining TFs: ASCL1, NEUROD1, POU2F3, and the fourth subtype. The nomenclature for the fourth subtype varies, referred to as YAP1,^189^ ATOH1,^190^ or Inflamed,^13^ depending on the specific SCLC model. SCLC–ASCL1/NEUROD1 were recognized as NE–SCLC, while SCLC–POU2F3/YAP1 is characterized as non‐NE–SCLC. The fourth subtype initially was characterized as YAP1 subtype. However, immunohistochemistry analysis in another literature failed to confirm a unique YAP1 subtype.^12^ In 2020, 38 circulating tumor cell‐derived explant models identified and validated a novel fourth SCLC subtype named SCLC–ATOH1.^190^ Importantly, distinct SCLC subtypes correlate with differential intratumoral immune status and, thus, are associated with distinct therapeutic sensitivities. In 2021, another study profiled the fourth subtype as SCLC‐inflamed (SCLC‐I) based on the immune infiltration status.^13^ Consistent with previous studies, SCLC‐I and POU2F3 express low or absent NE genes. Additionally, SCLC–YAP1 is validated to be enriched in T cell infiltration, whereas SCLC–ASCL1 and SCLC–NEUROD1 show less immune inflammation and T cell dysfunction.^191^, ^192^, ^193^ Non‐NE–SCLC exhibited higher MHC‐I expression than NE–SCLC type, achieving a durable immune checkpoint blockade response.^76^ Different SCLC molecular subtypes also exhibit distinct metastatic preferences. For example, SCLC–NEUROD1 is enriched in nodal and distant metastases, showing upregulation of EMT‐associated genes, TGF‐β, and STAT signaling.^193^ Thus, the distinct molecular subtypes of NE carcinomas have important clinical implications, including the potential for personalized treatment strategies, prognostic marker identification, and the development of new therapeutic approaches.

Somatic mutations and epigenetic alterations affect the aberrant regulation of various signaling pathways in NEtD including PI3K/AKT,^32^ JAK/STAT,^194^ FGFR,^194^ NOTCH,^57^, ^195^ Hippo/YAP1,^196^ and the DNA damage response pathway.^50^ Activation of PI3K/AKT,^32^ JAK/STAT,^194^ FGF/FGFR^194^ and inhibition of the NOTCH signaling are required for NEtD, while NOTCH and YAP activation inhibit NE cell fate.^144^, ^197^, ^198^ NOTCH pathway mutations were also observed in gastrointestinal NE carcinomas, especially in esophagus.^199^ In fact, multiple functional alterations affect more than a single pathway. Pathway alterations regulating NEtD are interconnected, co‐occurrent, or mutual exclusive,^200^ thus deeper research can provide more effective strategies for NEtD tumors treatment.

Of note, NEtD tumors also shares molecular features with hematological malignancies, indicating that existing blood cancer therapies can be applied to NEtD treatments.^4^, ^201^ Examples include XPO1 inhibitor and DNMT inhibitor. XPO1 inhibitor has been approved for treating multiple myeloma and can sensitize NEPC and SCLC to chemotherapy in preclinical models.^31^ DNMT inhibitor decitabine is US FDA approved for the treatment of acute and chronic leukemia, and it also exhibits improved effects for NEPC in combination with an ADC targeting B7‐H3 in preclinical findings.^67^

Meanwhile, disruption of membrane proteins patterns also drives NE lineage development through downstream signals, conferring them as potential biomarkers for diagnosis and therapeutic targets. Membrane protein‐targeted (DLL3) therapies, including antibody–drug conjugate and CAR, have gradually entered clinical trials for treatment‐limited NEtD tumors. Those secreted proteins (UCHL1 and XPO1) critical for mastering NE phenotype also can be a potential molecular indicator and therapeutic target for refractory NEtD tumors.

In conclusion, genetic, epigenetic alterations, abnormal TFs, and other dysregulated proteomics play a fundamental role in NE lineage plasticity, which drive targeted therapy resistance. We believe the clinical trials of genetic, epigenetic inhibitors, ADC drugs, and combination with existing standard chemotherapy or immunotherapy will demonstrate a promising antitumor efficacy and bring better outcomes for druggable NEtD tumors.

## AUTHOR CONTRIBUTIONS

All authors participated in the discussion of the draft. *Conceptualization, writing—draft and editing*: Jun Jiang and Donghui Han. *Supervision, writing—review and editing*: Rui Zhang, Weihong Wen, and Weijun Qin. *Visualization*: Jiawei Wang. All authors have read and approved the final manuscript.

## CONFLICT OF INTEREST STATEMENT

The authors declare that there is no conflict of interest.

## ETHICS STATEMENT

Not applicable.

## Data Availability

Not applicable.
